# Plant‐made dengue virus‐like particles produced by co‐expression of structural and non‐structural proteins induce a humoral immune response in mice

**DOI:** 10.1111/pbi.13501

**Published:** 2020-11-22

**Authors:** Daniel Ponndorf, Yulia Meshcheriakova, Eva C. Thuenemann, Albor Dobon Alonso, Ross Overman, Nicholas Holton, Stuart Dowall, Emma Kennedy, Martin Stocks, George P. Lomonossoff, Hadrien Peyret

**Affiliations:** ^1^ Department of Biological Chemistry John Innes Centre Norwich Research Park Norwich UK; ^2^ Leaf Expression Systems Norwich Research Park Norwich UK; ^3^ Public Health England Salisbury UK; ^4^ Plant Bioscience Limited Norwich Research Park Norwich UK

**Keywords:** virus‐like particles, dengue virus, Flavivirus, *Nicotiana benthamiana*, transient expression, antigen display, bluetongue virus CLPs

## Abstract

Dengue virus (DENV) is an emerging threat causing an estimated 390 million infections per year. Dengvaxia, the only licensed vaccine, may not be adequately safe in young and seronegative patients; hence, development of a safer, more effective vaccine is of great public health interest. Virus‐like particles (VLPs) are a safe and very efficient vaccine strategy, and DENV VLPs have been produced in various expression systems. Here, we describe the production of DENV VLPs in *Nicotiana benthamiana* using transient expression. The co‐expression of DENV structural proteins (SP) and a truncated version of the non‐structural proteins (NSPs), lacking NS5 that contains the RNA‐dependent RNA polymerase, led to the assembly of DENV VLPs in plants. These VLPs were comparable in appearance and size to VLPs produced in mammalian cells. Contrary to data from other expression systems, expression of the protein complex prM‐E was not successful, and strategies used in other expression systems to improve the VLP yield did not result in increased yields in plants but, rather, increased purification difficulties. Immunogenicity assays in BALB/c mice revealed that plant‐made DENV1‐SP + NSP VLPs led to a higher antibody response in mice compared with DENV‐E domain III displayed inside bluetongue virus core‐like particles and a DENV‐E domain III subunit. These results are consistent with the idea that VLPs could be the optimal approach to creating candidate vaccines against enveloped viruses.

## Introduction

Dengue virus (DENV) is a pathogenic virus that belongs to the family *Flaviviridae*. Four DENV serotypes (1–4) are currently recognized, all of which are transmitted by *Aedes* mosquitoes and which cause an estimated 390 million DENV infections per year (Bhatt *et al*., 2013). While the majority of infections are asymptomatic, DENV can also cause severe health problems including dengue haemorrhagic fever and dengue shock syndrome, which can be fatal (Halstead, 2007). DENV infection of cells occurs via receptor‐binding, uptake of the virus into the cell via endocytosis and release of the viral genome into the cytosol (Rodenhuis‐Zybert *et al*., 2010). The viral genome consists of a single strand of positive‐sense RNA, encoding a polyprotein containing three structural proteins (SP) and seven non‐structural proteins (NSP). The SPs include the capsid (C), the precursor membrane (prM) and the envelope (E) protein. Upon co‐translational translocation into the endoplasmic reticulum (ER), the polyprotein is proteolytically cleaved by host proteases (signal peptidases and furin) and the virus‐encoded non‐structural protein complex, NS2B‐NS3 (Hasan *et al*., 2018; Lindenbach, 2007). After cleavage of the polyprotein, the immature virions assemble in the ER lumen. NS2A recruits the viral RNA, structural proteins and the protease NS2B‐NS3 to the site of virion assembly and coordinates nucleocapsid and virion formation (Xie *et al*., 2019). After cleavage of the polyprotein, the nucleocapsid is formed and buds into the ER lumen. After assembly, the immature virions contain the nucleocapsid, prM, E and the host‐derived lipid membrane. Maturation occurs in the secretory pathway via the Golgi and the trans‐Golgi network by acidification‐dependent conformational changes and furin cleavage of prM. After the virion is released to the extracellular milieu, pr is released, resulting in a mature virion with an icosahedral‐like symmetry and a size of about 50 nm (Li *et al*., 2008).

Aside from traditional prevention of infection based on vector control, Sanofi Pasteur’s Dengvaxia (CYD‐TDV), a tetravalent vaccine based on chimaeric live yellow fever virus displaying DENV prM‐E, is licensed in 20 countries. However, it is controversial due to concerns regarding vaccine safety in young, seronegative patients due to antibody‐dependent enhancement (ADE) of infection (WHO, 2018). Because of this, much effort has been invested to improve the current DENV vaccine or to develop alternatives, in such a manner as to avoid triggering ADE and instead providing strong immune protection against all four serotypes. Strategies include the development of DNA vaccines, the use of live attenuated DENV and the production of DENV virus‐like particles (VLPs), DENV‐E domain III (EIII) subunits and the presentation of DENV‐EIII on chimaeric VLPs (Bhamarapravati and Sutee, 2000; Galula *et al*., 2014; Gottschamel *et al*., 2016; Kanagaraj *et al*., 2011; Metz *et al*., 2018; Pang *et al*., 2019; Ramasamy *et al*., 2018; Urakami *et al*., 2017; Zhang *et al*., 2011). As reviewed by Fuenmayor *et al*. (2017), out of all these strategies, the use of VLPs appears to be the most promising one because VLPs show advantages with regard to safety and efficacy compared with other strategies. In line with this, Boigard *et al*. (2017) showed for Zika virus, a close relative of DENV, that a VLP‐based candidate vaccine led to a higher titre of neutralizing antibodies in mice compared with an inactivated virus reference vaccine. The use of VLPs that contain the whole DENV‐E protein is further beneficial compared with EIII presentation, because production of neutralizing antibodies to a DENV infection is not limited to EIII in humans. Instead, binding of neutralizing antibodies to quaternary epitopes of the E protein has been described (de Alwis *et al*., 2012; Wahala *et al*., 2009).

Immunogenic DENV VLPs have previously been produced by expression of DENV prM‐E constructs in various expression systems such as mammalian cells (Metz *et al*., 2018; Urakami *et al*., 2017; Zhang *et al*., 2011), insect cells (Charoensri *et al*., 2014) and *Pichia pastoris* (Mani *et al*., 2013; Sugrue *et al*., 1997). Compared with these expression systems, the use of plants as a production platform for pharmaceutical products offers potential advantages regarding safety, cost‐effectiveness and appropriate post‐translational modifications (Lico *et al*., 2012; Ma *et al*., 2005; Ma *et al*., 2003; Twyman *et al*., 2003). Various non‐enveloped VLPs such as bluetongue virus (BTV) (Thuenemann *et al*., 2013), hepatitis B core antigens (HBcAg) (Mechtcheriakova *et al*., 2006; Peyret *et al*., 2015), norovirus (NV) (Mathew *et al*., 2014), poliovirus (PV) (Marsian *et al*., 2017) and nervous necrosis virus (NNV) (Marsian *et al*., 2019) have been successfully produced using transient expression in *Nicotiana benthamiana*. In the case of BTV, PV and NNV, the plant‐produced VLPs have been shown to be capable of providing protective immunity. There are fewer examples of the plant‐based production of VLPs of enveloped viruses, though enveloped influenza virus VLPs presenting haemagglutinin have been produced and represent the first plant‐based VLP candidate vaccine in phase III clinical trials (Medicago Inc. Quebec, Canada) (D'Aoust *et al*., 2008; Lomonossoff and D'Aoust, 2016).

In this work, we analyse the potential of plants for the production of enveloped DENV VLPs, using the pEAQ*‐HT* expression system (Sainsbury *et al*., 2009) in *Nicotiana benthamiana*. We compared the expression of a prM‐E construct, as used in other expression systems, with the co‐expression of structural protein (C‐prM‐E) and a NS5‐truncated version of NSP. For the first time, we could show that enveloped DENV1 VLPs can be purified after assembly in plants. The humoral immune response to DENV1 VLPs was analysed and compared with DENV‐EIII‐presenting BTV core‐like particles (CLPs) and a recombinant DENV‐EIII subunit in a mouse model. We show that VLPs induced a stronger B‐cell response than BTV‐CLPs displaying EIII and the EIII subunit alone, which is consistent with the idea that VLPs are more efficient than the other strategies in this regard.

## Results

**Figure 1:**
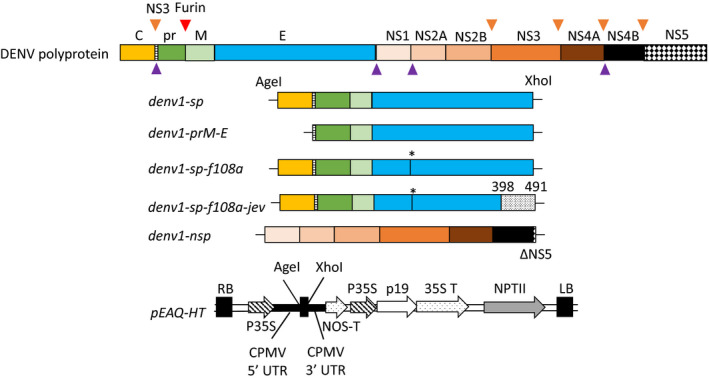
Organization of the DENV polyprotein and expression constructs used in this study (size of protein regions is not to scale). Top: Full DENV polyprotein containing the structural proteins (SP): capsid (C) in yellow, precursor membrane (prM) in green and light green and envelope (E) in cyan, and the non‐structural proteins (NSP) NS1 to NS5. Cleavage sites within the polyprotein are shown as triangles: NS3 = brown triangles, Furin = red and signal peptidase = purple. We expressed the full SP encoding the C, prM and E (*denv1‐sp*); a prM‐E construct (*denv1‐prM‐E)* containing the anchor region between C and prM (dashed region) and the NS3 cleavage site; *denv1‐sp‐f108a*, a version of SP carrying a F108A point mutation in the fusion loop of DENV‐E (*) and a version of *denv1‐sp‐f108a* in which we replaced the stem/anchor region of DENV‐E with the homologous sequence of Japanese encephalitis virus (grey shades in *denv1‐sp‐f108a‐jev*). Apart from *denv1‐prM‐E,* all constructs were co‐expressed with a NS5‐truncated version of the non‐structural proteins (NSP) (*denv1‐nsp*). For transient expression in *Nicotiana benthamiana,* all constructs were cloned into the pEAQ*‐HT* expression vector via *AgeI* and *XhoI* cloning sites (Sainsbury *et al*., 2009). RB: right border, LB: left border, p35S: cauliflower mosaic virus 35S promotor, CPMV: cowpea mosaic virus, UTR: untranslated region, NOS‐T: nopaline synthase terminator, p19: RNA silencing suppressor, 35S T: cauliflower mosaic virus 35 S terminator, neomycin phosphotransferase II (kanamycin resistance).

To produce DENV VLPs in *N. benthamiana,* we utilized two main strategies: expression of just a prM‐E construct (*denv1‐prM‐E*), which has been shown to lead to the formation of immunogenic subviral particles for different flaviviruses in various expression systems without the presence of NSP (Garg *et al*., 2017; Mason *et al*., 1991; Pincus *et al*., 1992; Takahashi *et al*., 2009), and the co‐expression of the whole SP (*denv1‐sp*) and a NS5‐truncated version of NSP (*denv1‐nsp*). The NS5 replicase gene was almost entirely deleted for biosafety reasons, to ensure that there was no risk of viral replication in any downstream experiment. For the second strategy, several constructs of *denv‐sp* were analysed (Figure 1). Along with the unmodified sequence, we introduced two modifications in the DENV‐E protein, which improved the secretion of DENV VLPs in other expression systems: a point mutation that replaces phenylalanine (F) with alanine (A) at position 108 in the fusion loop structure of the E protein (*denv1‐sp‐f108a*) as described by Urakami *et al*. (2017); and a combination of the point mutation and the replacement of the stem/anchor region of DENV1‐E with the corresponding sequence of Japanese encephalitis virus (JEV) (*denv1‐sp‐f108a‐jev*) (Chang *et al*., 2003; Hsieh *et al*., 2008).

### Expression analysis of DENV constructs

To analyse whether DENV proteins can be expressed in plants and are released into the total soluble protein (TSP) fraction, *N. benthamiana* plants were agroinfiltrated with the respective constructs (Figure 1) and harvested at 6 days post‐inoculation (dpi). The insoluble fraction (pellet) and the TSP were separated by centrifugation and analysed for the presence of prM and E by Western blot analysis with antibodies against prM and E, respectively.

**Figure 2:**
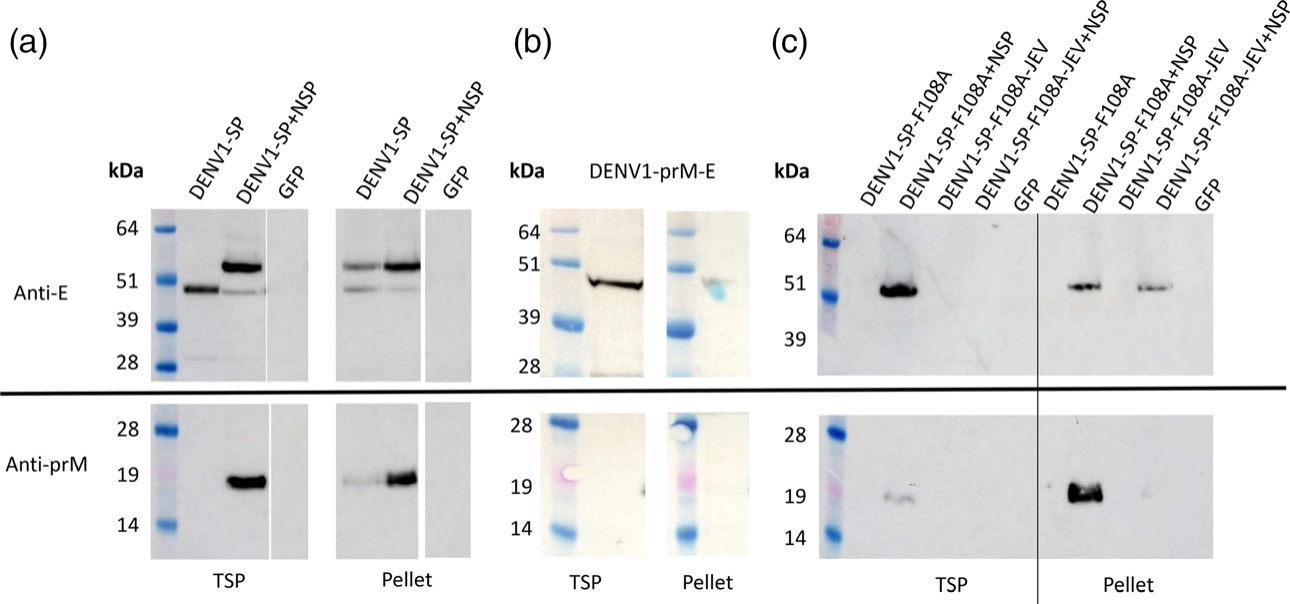
Western blot of DENV expression analysis in *N. benthamiana*. Uncropped versions of the Western blots are shown in Figure S2. Leaves were harvested 6 days post‐inoculation and analysed as described in Materials and methods. Primary antibodies against DENV‐E (upper row) and DENV prM (lower row) were used to detect the respective proteins. SeeBlue Plus2 pre‐stained protein standard was used as a marker. Band sizes are indicated in kDa. pEAQ‐*HT*‐*gfp* (*gfp)* was used as negative control. (a) Co‐expression of pEAQ‐*HT‐denv1‐sp* and pEAQ‐*HT‐denv1‐nsp*. The non‐structural proteins are required to release prM (~18 kDa) in the soluble fraction. E occurs at 55 and 49 kDa. The 55 kDa size is only visible in the TSP after co‐expression of pEAQ‐*HT‐denv1‐nsp*, indicating aberrant processing in the absence of NSP. (b) pEAQ‐*HT‐denv1‐prM‐E* led to no detection of prM and the release of E in the TSP in the 49 kDa form. (c) Vertical line within blot separates TSP and pellet fraction for better visualization. PrM and a 55 kDa version of E can be detected in the TSP after co‐expression of pEAQ‐*HT‐denv1‐sp‐f108a* and pEAQ‐*HT‐denv1‐nsp*. For pEAQ‐*HT‐denv1‐sp‐f108a‐jev,* no proteins could be detected in the TSP, indicating a negative influence of the JEV sequence on SP solubility.

As shown in Figure 2, we observed different expression patterns between the individual constructs and construct combinations that were mainly influenced by the presence of the non‐structural proteins. Figure 2a shows that the prM moiety is only detected in the total soluble protein fraction when *denv1‐nsp* is co‐expressed with *denv1‐sp*. Moreover, we detected two sizes of DENV‐E of about 49 and 55 kDa, respectively (Figure 2a). The expected size of the glycosylated E protein is ~55 kDa, so we hypothesized that the 49 kDa form might be a non‐glycosylated version of E, or a degradation product. Because the 55 kDa version could be detected in the TSP only after co‐expression of *denv1‐nsp*, incorrect processing of the E protein in the absence of NSP seems likely. This might also be the case for *denv1‐prM‐E* (Figure 2b), which was not co‐expressed with *denv1‐nsp* and led to the detection of only the 49 kDa fragment of E, and no detection of prM at all. By contrast, the prM moiety could be detected in the TSP after expressing *denv1‐sp‐f108a* in the presence of *denv1‐nsp*, but not after expressing *denv1‐sp‐f*10*8a‐jev,* regardless of the presence of NSP (Figure 2c). The detected size of 18 kDa in all cases where prM is detected indicates that this protein is not cleaved to pr and M (each ~9 kDa) in plants, most likely due to the lack of furin in *N. benthamiana* (Wilbers *et al*., 2016).

In contrast to prM, the E protein could be detected for all constructs expressed in the presence of NSP, though size and solubility of the detected E protein varied between E‐expressing constructs. Without co‐expression of *denv1‐nsp*, we could not detect the E protein from expression of *denv1‐sp‐f108a* or *denv1‐sp‐f108a‐jev* (Figure 2c). While the presence of NSP led to the release of E into the TSP for *denv1‐sp‐f108a*, the protein remained insoluble for *denv1‐sp‐f108a‐jev*, indicating a negative influence of the JEV sequence on protein processing and solubility in plants. Uncropped images of the original Western blots shown in Figure 2 can be found in Figure S2.

These results show that DENV1 structural proteins can be expressed successfully in plants and that the presence of NSP has a substantial influence on the SP. This is consistent with NS3 and NS2B (at least) being processed and proteolytically active in plants, because the activity of NS3 depends on the presence of NS2B (Falgout et al., 1991, Perera and Kuhn, 2008).

Since the expression of full‐length SP together with NSP gave the most promising results at this stage, we analysed the same strategy with DENV serotypes 2 and 4. However, we could not detect any protein expression for either serotype (Figure S1).

### Purification and characterization of DENV1 VLPs

**Figure 3:**
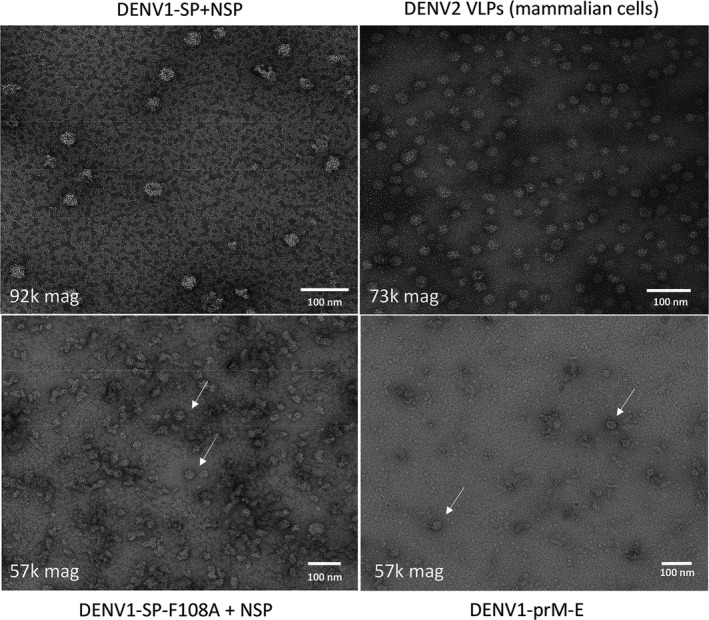
TEM analysis of purified samples: samples were negatively stained with 2% (w/v) uranyl acetate as described in Materials and methods. Magnifications of pictures is shown on the bottom left. Scale bars on the bottom right are 100 nm each. Particles for DENV1‐SP + NSP (top left) look comparable to a DENV2 VLP‐positive control (top right). DENV1‐prM‐E and DENV1‐SP‐F108A + NSP were less convincing due to the presence of plant‐derived impurities, but VLP‐like structures are visible in the sample (white arrows).

To determine whether the expression of *denv1‐prM‐E* and the co‐expression of *denv1‐nsp* with *denv1‐sp* or *denv1‐sp‐f108a* led to the formation of VLPs, a three‐step ultracentrifugation‐based purification protocol (shallow sucrose gradient, glycerol gradient and long sucrose gradient) was developed as described in Materials and Methods. Transmission electron microscopy (TEM) analysis with negative staining revealed the assembly and formation of VLPs after co‐expression of *denv1‐sp* and *denv1‐nsp*. These particles varied in size from 25 to 40 nm and looked comparable to a DENV2 VLP‐positive control produced in mammalian cells (Figure 3), which showed a similar variation in size. The size of the VLPs and the presence of uncleaved prM suggest that the purified VLPs did not undergo the maturation process. TEM analysis of DENV1‐prM‐E showed more impurities and fewer particles than DENV1‐SP. The DENV‐SP‐F108A‐JEV samples showed a high background of plant‐derived impurities and the presence of VLPs could not be confirmed unequivocally, though some VLP‐like structures were seen (Figure 3).

**Figure 4:**
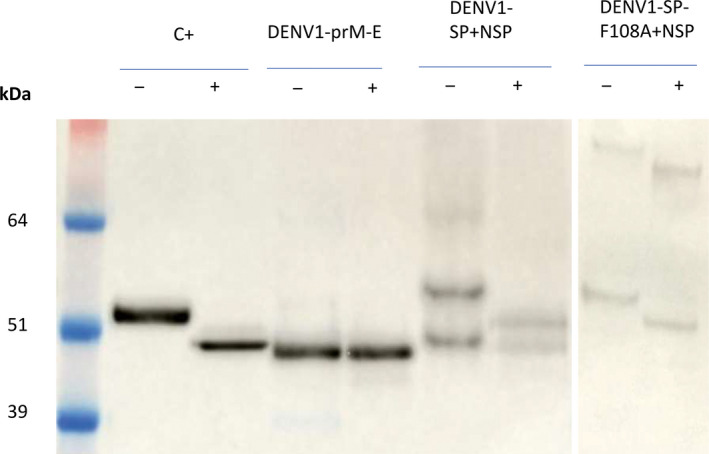
Deglycosylation of DENV1‐VLPs using PNGase F. Samples were treated under non‐denaturing conditions with (+) and without (−) PNGase F for 24 h at 37 °C. Size shift of bands indicates removal of N‐glycans at position N67 and/or N167 of the E protein. The expected size of the deglycosylated E protein is about 49 kDa. C+: Commercial DENV2 VLPs produced in mammalian cells.

To further characterize the VLPs, we used PNGase F to determine whether the purified particles are glycosylated. DENV‐E protein contains two N‐linked glycosylation sites at positions N67 and N167. Mondotte *et al*. (2007) showed that deglycosylation of either site leads to a visible size shift in a Western blot (Mondotte *et al*., 2007). We estimated the expected size shift of deglycosylation from 55 kDa to about 49 kDa using a mammalian cell‐produced VLP‐positive control (Figure 4). Treatment with PNGase F led to a size shift in the control and for DENV1‐SP‐F108A, but not for DENV1‐prM‐E, which indicates that the E protein is not glycosylated in the latter case. For DENV1‐SP, the 55 kDa E band shows a shift to about 50 kDa after PNGase F treatment, while the 49 kDa band retains the same size. This indicates that the co‐expression of *denv1‐sp* and *denv1‐nsp* results in VLPs that contain a mixture of glycosylated and non‐glycosylated E protein. A proteolytically cleaved version of the E protein that is not susceptible to deglycosylation because of loss of N67 and N167 would have an estimated size of 37 kDa. Therefore, it is unlikely that the 49 kDa is a product of proteolysis.

The size difference between the commercial, mammalian cell‐derived DENV control and the plant constructs before treatment is likely caused by differences in glycosylation between the two expression systems.

### Antigen display inside of bluetongue virus core‐like particles and design of an E domain III subunit

**Figure 5:**
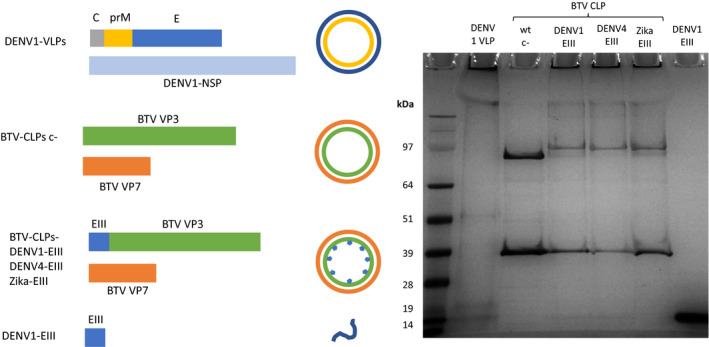
Analysed candidate vaccines and Coomassie‐stained SDS‐PAGE control: The respective vaccine candidates were expressed in plants, purified and analysed by SDS‐PAGE. DENV1‐VLPs were produced by co‐expressing DENV1‐SP and NSP. SDS‐PAGE shows signals for prM (18 kDa) and E (~55 kDa). Empty bluetongue core‐like particles (BTV‐CLPs) as negative control (c‐) were produced by co‐expression of BTV VP3 (103 kDa) and VP7 (39 kDa). For antigen display inside BTV‐CLPs, the E domain III (EIII) of DENV1, DENV4 and ZIKA was fused to the *N* terminus of BTV‐VP3. Co‐expression of BTV‐VP3 and V7 leads to assembly of CLPs and integration of the *N*‐terminal fused antigens. The Coomassie‐stained SDS‐PAGE shows the size difference between c‐ VP3 and VP3 fused to EIII. The EIII soluble subunit (14 kDa) showed the expected size of 14 kDa and high purity.

Since we could not produce VLPs for other DENV serotypes, and presentation of a DENV‐EIII domain on hepatitis B core antigen VLPs previously led to antibody production in mice (Pang *et al*., 2019), we analysed EIII display as a possible alternative to a VLP approach. In an attempt to provide improved T‐cell‐based immunity, packaging of the antigen on the inner surface of a VLP was explored, as the antigen should be protected until autophagy by antigen‐presenting cells, thereby allowing efficient presentation. Therefore, we displayed the EIII ectodomain of DENV1, DENV4 and Zika virus on the inner surface of BTV core‐like particles (B‐CLPs) by *N*‐terminal fusion of the antigen to bluetongue VP3 (Thuenemann and Lomonossoff, 2018, Charpilienne *et al*., 2001). Unmodified B‐CLPs and B‐CLPs presenting the EIII domain of DENV1, DENV4 and Zika were expressed in plants, and purified and analysed by TEM, SDS‐PAGE and mass spectrometry. We could show that integration of the EIII did not prevent VLP assembly (Figure S3) and confirmed integration of the respective antigens by mass spectrometry (Figure S4) and a visible size shift of VP3 in SDS‐PAGE (Figure 5).

To compare the VLP approach to a soluble subunit antigen strategy, we also produced a histidine‐tagged DENV1 EIII subunit. The peptide was translocated to the ER by using the transit peptide of *Arabidopsis thaliana* basic chitinase and a C‐terminal KDEL sequence for ER retention. The protein was expressed in *N. benthamiana*, purified by immobilized metal affinity chromatography and analysed by SDS‐PAGE (Figure 5).

### Antigenicity of DENV1‐VLPs

To determine whether the purified DENV1 VLPs are immunologically active, we conducted antigenicity assays in 6‐to 9‐week‐old BALB/c mice using the vaccine candidates shown in Figure 5.

Six mice per group were immunized in a prime‐boost manner (0 and 14 days) for all tested vaccine candidates. Additionally, DENV1‐VLPs, B‐CLP‐DENV1‐EIII, B‐CLP‐DENV4‐EIII and B‐CLP‐ZIKV‐EIII were analysed in a single immunization (day 0), resulting in a total of 10 tested groups. For the VLPs/B‐CLP groups, a 10 µg/dose was applied, while for the EIII subunit, the concentration was adjusted to 0.7 µg/dose to analyse comparable molar amounts of delivered antigen. All candidates were applied without adjuvants.

Bodyweight and temperature were monitored over 28 days as indicators for animal health. No evidence of weight loss or any temperature fluctuations were detected, indicating that animals remained healthy throughout the experiment.

**Figure 6:**
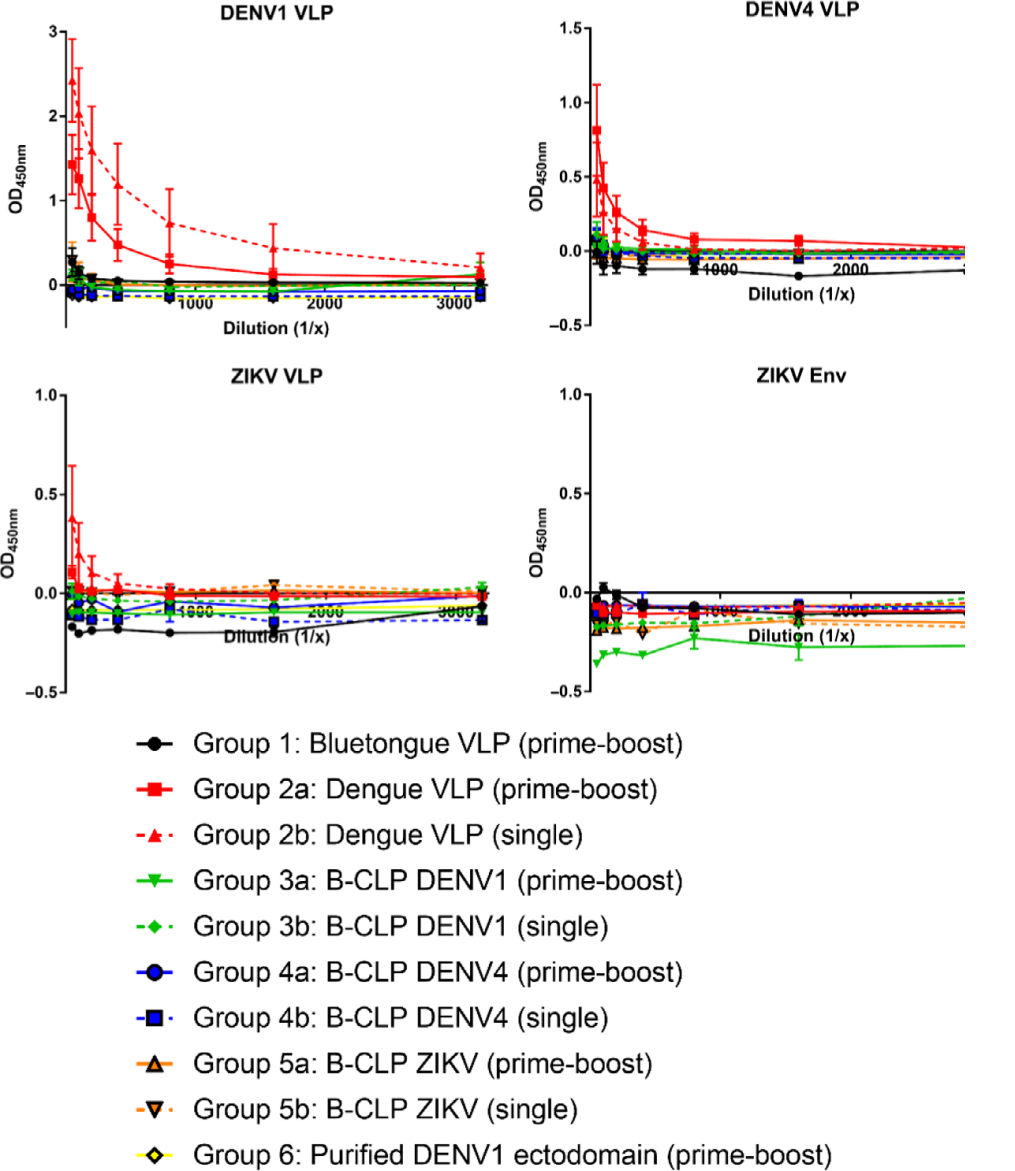
Humoral response of DENV1 VLPs, EIII domains displayed in bluetongue CLPs and the DENV1‐EIII subunit. Six mice per group were immunized in a single or prime‐boost manner. Collected sera of immunized mice were tested in a dilution series against commercial DENV1, DENV4 and ZIKA VLPs, as well as recombinant ZIKA envelope protein (ZIKV‐Env). Lines show mean values with error bars denoted standard error. Only DENV1‐VLPs stimulated the production of reacting antibodies, indicating that in mice, VLPs are more efficient antigens in terms of B‐cell response than comparable amounts of the DENV1 subunit alone or the display of EIII on bluetongue CLPs.

To detect virus‐specific antibody responses, ELISA plates were coated with mammalian cell‐produced DENV1, DENV4 and Zika virus (ZIKV) VLPs and ZIKV E protein. The mouse serum samples were applied and analysed for antibody binding to the antigen. As shown in Figure 6, only sera collected from mice immunized with DENV1‐VLPs showed specific binding to DENV1 antigen. Some cross‐reactivity was observed to DENV4 and ZIKV VLPs, but not to ZIKA E. Surprisingly, the antibody response after a single immunization was stronger compared with the prime‐boost group, although this was not statistically significant and might result from the small number of mice per group. These results indicate that VLPs are more efficient B‐cell response activators in mice compared with comparable doses of the DENV1 subunit and the display of EIII within BTV CPLs.

## Discussion

The aim of this study was to analyse the suitability of plants as a production platform for flaviviral VLPs. Immunogenic DENV VLPs have been produced in mammalian cells (Metz *et al*., 2018; Urakami *et al*., 2017; Zhang *et al*., 2011), insect cells (Charoensri *et al*., 2014) and yeast (Mani *et al*., 2013; Sugrue *et al*., 1997). These VLPs were produced using DENV prM‐E constructs, which led to the assembly of VLPs in the absence of non‐structural proteins. This strategy was also analysed by Kanagaraj *et al*. (2011) for DENV3 VLPs in lettuce chloroplast, and the expression of the E protein was shown, but the data analysing the assembly of DENV3 VLPs were not conclusive. In contrast to the approach using just prM‐E constructs in other systems, our results show that this strategy was not successful in *Nicotiana benthamiana*. To achieve VLP assembly, glycosylation and solubility in plants, the co‐expression of DENV‐NSP was necessary. This indicates that protein processing in plants for DENV constructs differs compared with other expression systems. This is further underlined by the observation that the replacement of the stem/anchor region of DENV1‐E with the sequence of JEV seemed to prevent VLP secretion in plants instead of promoting it as described in mammalian cells (Chang *et al*., 2003; Hsieh *et al*., 2008). Despite the different expression strategy, our plant‐made VLPs showed similarities in size and appearance to VLPs expressed in mammalian cells and led to B‐cell activation and antibody production in mice. Furthermore, as observed in the current study, Hsieh *et al*. (2008) described the presence of two sizes of DENV‐E. Our data suggest that this might be caused by a mixture of glycosylated and non‐glycosylated E protein. At this point, we can only speculate what causes the mix in glycosylation pattern, but once we integrated the F108A mutation in the E protein, we could only detect the glycosylated size of E. It might be possible that the F108A mutation in the fusion loop of E promotes secretion as described by Urakami *et al*. (2017) influencing the glycosylation efficiency of VLPs in plants. However, we did not observe an increase in VLP yield caused by F108A but instead faced more difficulties separating these VLPs away from plant impurities, which makes it difficult to definitively confirm that this mutant allows assembly into VLPs in plants.

As described for mammalian cell‐expressed DENV VLPs (Metz *et* *al*., 2018; Urakami *et al*., 2017), we observed that the prM protein was not cleaved into pr and M, which means that the VLPs are not matured and most likely present prM on the VLP surface (Li *et al*., 2008). For plant‐made VLPs, this observation was not surprising, because plants do not produce furin (Wilbers *et al*., 2016), the protease that cleaves prM during viral maturation (Li *et al*., 2008). However, the presence of prM on the VLP surface could be problematic for a vaccine candidate, because anti‐prM antibodies are non‐neutralizing and enhancing (Smith *et al*., 2016). A solution for this problem could be the co‐expression of a recently designed human furin variant, which showed high *in vivo* activity in plants (Mamedov *et al*., 2019).

A potential VLP‐based DENV vaccine must contain all four serotypes of DENV to avoid the potential of ADE. Because the expression of DENV3 prM‐E constructs was previously shown in lettuce chloroplasts (Kanagaraj *et al*., 2011), we initially tried to produce VLPs for DENV2 and 4. Surprisingly, we could not detect any protein expression for either serotype. We interpret this as being caused by differences in the efficiency of expression and conformational folding of the DENV proteins between various sero‐ or even genotypes, since radical differences in plant‐produced viral protein yield between different serotypes of the same virus has been observed on numerous occasions: notably, poliovirus (Marsian, 2016) and bluetongue virus (Fay, 2018). This should be considered for further work in this field, because finding a suitable genotype of the respective serotypes for expression in plants is likely to be a key determinant of recovered yield. Had this project gone differently, we would have compared VLPs of the four different DENV serotypes, but we were only able to consistently express VLPs for DENV‐1. It should be noted that DENV‐1 VLPs concentrated the majority of our efforts in terms of optimizing construct design and purification protocol, so it is possible that more time and resources would have allowed us to obtain VLPs of the other serotypes as well. Understanding and correcting the causes for differing yield between these serotypes would allow for the development of a plant‐produced tetravalent vaccine.

We were however able to test a range of strategies, and so antigen display and a subunit vaccine strategy were tested as potential alternatives to VLPs. We designed bluetongue core‐like particles (B‐CLP) carrying DENV1, DENV4 and Zika EIII antigens on the inside of the particle. Of the many strategies investigated to obtain VLP‐based flavivirus vaccine candidates in plants (most of which are not discussed here), this approach was by far the most successful in terms of recovered yield and ease of purification. Indeed, BCA assay quantification of the DENV VLP vaccine preparation indicated that purified recovered yield was about 2 µg of VLP per gram of fresh‐weight‐infiltrated leaf tissue (FWT) after three successive ultracentrifugation steps and multiple buffer exchange/concentration steps, while the B‐CLPs yielded 5–15 µg/g FWT after just one ultracentrifugation step and a single buffer exchange/concentration step. We therefore reasoned that these B‐CLPs were worth testing in mice despite the fact that they represent an unorthodox approach to antigen presentation (with the antigen 'presented' on the inside of the VLP); however, we hypothesized that this approach may result in improved T‐cell responses. This decision was supported by the publication of a similar approach using GFP fused to the inside of rotavirus particles, to elicit an antibody response to GFP (Charpilienne *et al*., 2001), although that study used adjuvant and higher inoculum doses compared with the protocol used here. As an alternative, we could have included tandem hepatitis B core‐based CLPs displaying DENV‐EIII on their outer surface (as described in Pang *et al*., 2019) for the mouse immunization studies, but preferred to analyse the more novel B‐CLP candidates.

While DENV1 VLPs induced an antibody response in mice, none of the DENV‐B‐CLPs showed any effect at the doses used. In line with this, serum from mice immunized with B‐CLP‐Zika EIII did not lead to any detectable antibody response when tested against the commercial Zika envelope protein. Antigen display of DENV‐EIII domain on mosaic hepatitis B surface antigens (Ramasamy *et al*., 2018) and on hepatitis B core antigen VLPs (Arora *et al*., 2013; Pang *et al*., 2019) previously led to an immune response in mice. We therefore conclude that B‐CLP is a poor carrier for DENV and Zika EIII domain antigens in terms of B‐cell activation in mice. DENV1 VLPs were also superior compared to the EIII domain subunit antigen, which was not unexpected, because VLPs are usually more efficient due to their size and repetitive surface (Bachmann and Jennings, 2010; Bachmann and Zinkernagel, 1997; Link *et al*., 2012). We did not observe any increase in antibody titres in the mice that received a booster of DENV1 VLPs compared with those that received only the prime. In fact, antibody titres from boosted animals were lower than in those that were only primed, although this difference was not statistically significant. It is not clear why this might be the case, but there are examples of booster vaccinations (with live attenuated virus vaccines) having limited effect on the stimulation of the immune response (Christenson and Bottiger, 1994; Kongsgaard *et al*., 2017). We also carried out interferon‐gamma ELISPOT assays on the splenocytes of vaccinated mice, and the results of these suggest that there may be a specific T‐cell response stimulated by the B‐CLPs (but not by the DENV1 VLPs) after a prime‐boost regimen; however, the results were not statistically significant, so no firm conclusions could be drawn from these experiments (Figure S6).

For further studies, it would be interesting to directly compare the neutralizing antibody response of DENV1 VLPs with E‐DIII displaying hepatitis B particles, because the production of neutralizing antibodies in humans is not limited to the EIII region but rather involves the quaternary structures formed by E protein multimers (de Alwis *et al*., 2012; Wahala *et al*., 2009). Furthermore, the extension of the EIII domain subunit with a part of the E domain II led to improved neutralizing antibody titre (Park *et al*., 2020), indicating that the use of VLPs displaying the whole E protein is likely to be the most suitable DENV vaccine strategy, provided that prM cleavage and maturation of VLPs can be achieved to avoid the production of non‐neutralizing, but enhancing antibodies. In fact, we attempted to display the entire DENV1 E ectodomain on the surface of tandem hepatitis B core particles to compare this external antigen display strategy with our DENV1 VLPs, but we were unable to produce such full‐length E‐displaying HBcAg CLPs.

This work joins a very small number of published examples of plant‐produced enveloped VLPs that could be purified and shown to be immunogenic. The only other examples to our knowledge are influenza virus haemagglutinin (HA) VLPs (D'Aoust *et al*., 2010; D'Aoust *et al*., 2008) and chimaeric VLPs composed of the ectodomain of Rift Valley fever virus Gn fused to the transmembrane and cytosolic domains of influenza HA (Mbewana *et al*., 2019). Our work therefore represents the first example of such plant‐produced enveloped VLPs in which influenza HA is not used to supply the transmembrane domain of the viral glycoprotein. At present, plants are a challenging expression system for these types of VLPs, because of differences in protein processing compared with other expression systems and challenging product purification compounded by low yield. However, if a more suitable purification system is established and we gain more insights into the protein processing of DENV proteins in *N. benthamiana*, plants could one day prove to be a highly scalable and cost‐efficient expression platform, as they are for influenza VLPs.

## Methods

### Construction of plant expression vectors

Coding regions of the structural (SP) and a NS5‐truncated version of non‐structural proteins (NSP) of DENV serotype 1 (strain Hawaii, GenBank: KM204119.1), serotype 2 (Strain New Guinea C, GenBank KM204118.1) and serotype 4 (Strain H241, GenBank KR011349), and the structural proteins of Zika virus (strain ZikaSPH2015, GenBank KU321639) were codon‐optimized for expression in *N. benthamiana* and synthetized by GeneArt (Life Technologies Ltd., United Kingdom). The prM‐E sequence (*denv1‐prM‐E*) was amplified from *denv1‐sp* using primers *denv1‐prM‐E*‐fw and *denv1‐prM‐E*‐rv (Table S1). The F108A point mutation, replacing a phenylalanine at position 108 of the DENV‐E protein with alanine (Urakami *et al*., 2017), was inserted using the GeneArt site directed mutagenesis kit (Invitrogen™, USA) into *denv1‐sp* according to the manufacturer’s protocol using primers *denv1‐f108a*‐fw and *denv1‐f108a*‐rv (Table S1) resulting in *denv1‐sp‐f018a*. The stem/anchor region of DENV1‐E (amino acids 398‐491) in *denv1‐sp‐f108a* was replaced with the corresponding sequence of JEV (strain Nakayama, GenBank: EF571853.1) according to Chang *et al*. (2003) and Hsieh *et al*. (2008) resulting in *denv1‐sp‐f108a‐jev*. E domain III fusions to BTV VP3 were made by amplifying *denv1‐sp* with primers *DENV1‐EIII‐fw* and *DENV1‐EIII‐rv*, *denv4‐sp* with primers *DENV4‐EIII‐fw* and *DENV4‐EIIID3‐rv,* and with primers *ZIKV‐EIII‐fw* and *ZIKV‐EIII‐rv* (Table S1). All amplified E domain III fragments were flanked by NruI/AgeI and BspEI restrictions sites for cloning into pEAQ‐GFP:VP3 (Thuenemann and Lomonossoff, 2018). The EIII subunit was amplified from pEAQ‐*HT*‐DENV1‐SP using primers DENV1‐EIII‐fw and rv (Table S1). For targeting to the ER, an N‐terminal transit peptide from *Arabidopsis thaliana* basic chitinase (MKTNLFLFLIFSLLLSLSSAE) was amplified to DENV1‐EIII using primer TP‐Atl‐fw. For purification, a 6xHis‐tag was amplified using primer DENV1‐EIII‐his‐rv.

All coding regions were flanked by AgeI and XhoI restriction sites and cloned into the similarly digested pEAQ‐*HT* expression vector (Peyret and Lomonossoff, 2013, Sainsbury *et al*., 2009). Plasmids were transformed into *E.coli* top 10 cells (Life Technologies Ltd, Thermo Fisher Scientific, UK) for plasmid propagation, and sequences were confirmed by sequencing. All plasmids were transformed into *Agrobacterium tumefaciens* LBA4404 (or AGL1 for pEAQ‐*HT‐denv1‐EIII*) for transient expression in *N. benthamiana*.

### Protein expression and analysis

Transient expression in 3‐ to 4‐week‐old *N. benthamiana* plants was carried out by agroinfiltration as described by Thuenemann *et al*. (2013). For B‐CLPs, constructs for EIII:VP3 fusion proteins were co‐infiltrated with pEAQ‐VP7*HT* (Thuenemann *et al*., 2013). Leaves were harvested 6 days post‐infiltration (dpi) and analysed for protein expression modified from Peyret *et al*. (2015). A total of 6 leaf discs were punched out of the leaves using a cork borer (number 6, Sigma‐Aldrich Co. Ltd., catalogue number Z165220). The discs were homogenized in 270 µL NPI buffer (50 mm sodium phosphate, 300 mm NaCL, pH8.0, EDTA‐free complete protease inhibitor cocktail (Roche Diagnostics GmbH, Germany)) using a Bead Ruptor 24 (Camlab, United Kingdom, speed = 4, 30 s, 5 °C). Homogenized samples were centrifuged (10 min, 16 000 ***g***, 5 °C), and supernatant was collected and clarified a second time under the same conditions. The pellet was resuspended in 200 µL NPI buffer. Pellet and clarified supernatant were mixed in a 1:1 ratio with loading buffer, which consisted of a 3:1 ratio of NuPAGE LDS buffer (Novex, Life Technologies, Carlsbad, CA) and 2‐mercaptoethanol. Samples were then boiled for 20 min and clarified for 10 min at room temperature (16 000 ***g***).

Samples were analysed by electrophoresis on a 4%–12% (DENV‐E) or 12% (DENV prM) NuPAGE Bis‐Tris polyacrylamide gels (Novex, Life Technologies). Gels were stained using InstantBlue™ (Expedeon Ltd., UK) or analysed by Western blot as follows: proteins were blotted on a nitrocellulose membrane using the Trans‐Blot Turbo™ RTA Transfer Kit in a Trans‐Blot Turbo™ System (Bio‐Rad). Membranes were blocked overnight in blocking buffer (5% (w/v) skimmed milk powder in phosphate‐buffered saline with 0.1% (v/v) Tween‐20). Primary antibodies were used in a 1:2000 dilution in blocking buffer for 2 h at room temperature (RT): anti‐DENV‐E: mouse, monoclonal (cat. number: MCA2277, Bio‐Rad, United Kingdom); and anti‐DENV prM: rabbit polyclonal (cat. number: GTX128093, Insight Biotechnology Ltd., United Kingdom). The secondary antibodies were diluted 1:10 000 in blocking buffer and incubated for 1 h at RT: anti‐rabbit (Amersham™, goat anti‐rabbit IGG, HRP, cat. number: RPN4301GE Healthcare, United Kingdom) and anti‐mouse (HRP‐goat anti‐mouse, cat. number: 626520, Life Technologies). For development, chemiluminescent substrate (Immobilon™, Millipore, Burlington, MA) was used and chemiluminescence was detected using an ImageQuant LAS 500 (GE Healthcare UK Ltd., United Kingdom).

### Isolation of DENV1 virus‐like particles from plant material

Leaves were harvested at 6 dpi and blended in 3 volumes of TNE buffer (10 mm Tris‐HCl, 120 mm NaCl, 1 mm EDTA, EDTA‐free complete protease inhibitor cocktail (Roche Diagnostics GmbH, Germany), pH = 8.0). Crude extract was filtered through one layer of Miracloth (Merck KGaA, Germany) and clarified (30 min, 4 °C, 29 000 ***g***). The supernatant was filtered through a 0.45‐µm filter (Sartorius GmbH, Germany) and loaded on a discontinuous 10%–70% (w/v) sucrose gradient (10% steps, 2 mL each fraction, sucrose prepared in TNE buffer). Fractions were collected from the bottom in 2 mL steps after 4‐h centrifugation in a SureSpin 630/36 rotor (Thermo Fisher Scientific) at 167 000 ***g***, 4 °C, 4 h, and analysed by Western blot for the presence of DENV‐E. The 40% fraction was diluted 1:1 with TNE and loaded on a discontinuous glycerol gradient (2 mL fractions; 10%, 15%, 20%, 30% (v/v) gradient; glycerol diluted in TNE buffer). These gradients were centrifuged in a TH641 rotor (Thermo Fisher Scientific) at 274 000 ***g***, 4 °C, 4 h. Fractions were collected from the top of the gradient in 2 mL steps. The DENV‐E‐positive fractions were determined by Western blot, pooled and concentrated to a final volume of 2 mL using Amicon Ultra‐15 100 000 MWCO spin concentrators (Merck Millipore Ltd., Ireland) as described by the manufacturer. To remove residual glycerol, the buffer was exchanged to TNE during concentration. For the final purification, the concentrated samples were loaded on a second discontinuous sucrose gradient (20%–60% (w/v)) and centrifuged at 274 000 ***g***, 4 °C, 3 h in a TH641 rotor (Thermo Fisher Scientific). The gradients were fractionated from the top and analysed by Western for the presence of DENV‐E. Figure S5 shows the Western blots used to decide which fractions of the three different gradients to take forward in the purification. Positive fractions from the final gradient were concentrated to a final volume of about 2 mL as described before, and sucrose was removed during concentration by buffer exchange with phosphate‐buffered saline (PBS)‐G (PBS 100, Formedium, UK, pH = 7.4, 10% (v/v) glycerol). Samples were snap‐frozen in liquid nitrogen and stored at −80°C until further analysis by SDS‐PAGE, BCA assay protein quantification (Thermo Fisher Scientific) and transmission electron microscopy.

### Purification of B‐CLPs

Leaf tissue was harvested at 8 dpi and blended in 3 times the volume of BTV CLP extraction buffer (50 mm Bicine, pH 8.4, 140 mm NaCl, 1% (w/v) NLS [N‐lauroylsarcosine sodium salt], 1 mm DTT, 0.5 × Complete EDTA‐free Protease Inhibitor) using a Waring blender. The homogenized material was filtered through 2 layers of Miracloth and clarified by centrifugation (13 000 ***g***, 10 °C, 10 min).

The clarified supernatant was loaded on an Optiprep gradient (20%, 30%, 40%, 50%, 3 mL per fraction). Optiprep was prepared in 6× CLP purification buffer (300 mm Tris‐HCl, pH8, 840 mm NaCl, RT). Gradients were centrifuged in a SureSpin 630/36 rotor (Thermo Fisher Scientific) at 85710 *g*, 10 °C, 3 h. 7 fractions (first fraction 2 mL, fraction 2–7 1 mL each) were collected from the bottom of the tube, and 6 µL of each fraction was analysed by SDS‐PAGE to identify fractions containing CLPs. Figure S5 shows the gel used to decide which fractions to take forward. These were concentrated to a final volume of about 2 mL, and Optiprep was removed during concentration by buffer exchange with phosphate‐buffered saline (PBS)‐G and characterized/stored as described above.

### Purification of DENV1‐EIII

Plants were harvested at 4 dpi and blended in 3 times extraction buffer (50 mm Tris, 300 mm NaCl, 1 mm PMSF, 10 mm Na metabisulphate, pH 7.0) at medium speed for 40 s 4 times using a Waring blender. The homogenate was filtered using a 1‐µm bag filter (spectrum) and centrifuged at 4000 ***g*** for 15 min and 10 000 ***g*** for 15 min at 4 °C. Supernatant was filtered in 3 steps through a 1‐µm to 0.8‐µm and 0.22‐µm PolyCap filter (Whatman GE) followed by a 0.22‐µm PES bottle top filter (Nalgene) before immediately loading onto a 5 mL HiTrap Talon Crude column equilibrated in 50 mm Tris, 300 mm NaCl, pH 7.0 using an AKTA Avant (GE Healthcare). The column was washed as follows: 20 column volumes (CVs) of equilibration buffer, 10 CVs of wash buffer (50 mm Tris, 300 mm NaCl, 0.1 % Triton X‐100, pH 7.0) and 10CVs of equilibration buffer. Protein was eluted with 10CVs of elution buffer (50 mm Tris, 300 mm NaCl, 200 mm imidazole, pH 7.0.). The flow‐through sample was reloaded and the purification repeated to ensure that all material present was captured. Fractions from containing target protein, as confirmed by SDS‐PAGE, were pooled and concentrated using a PES 10k MWCO centrifugal concentrator (Millipore) before applying at 1 mL/min to a 120 mL Superdex 75 column (GE Healthcare) that had been pre‐equilibrated in gel filtration buffer (50 mm Tris, pH 7.0, 140 mm NaCl). Fractions containing target protein were pooled, 0.22 µm sterile‐filtered and concentrated using a 10 kDa MWCO centrifugal concentrator (Millipore) to 0.85 mg/mL.

For immunogenicity assays, all samples were buffer‐exchanged to PBS‐G and quantified using the Pierce BCA Protein Assay Kit (Thermo Fischer Scientific) according to the manufacturer’s protocol to calculate doses for animal experiments.

### Negative‐stain transmission electron microscopy (TEM) and particle measuring

Samples were adsorbed on a 400 mesh carbon‐coated copper grid (EM resolutions UK) for 30 s. The grids were washed with 8 drops of H_2_O_dd_ and stained with 2% (w/v) uranyl acetate for 15–30 s and imaged using a Talos F200C TEM fitted with a Gatan OneView camera. Particles were measured using the ImageJ software for Windows.

### Deglycosylation of DENV VLPs

Glycerol‐free PNGase F (New England Biolabs Inc., USA) was used according to the manufacturer’s protocol under native conditions with an incubation time of 24 h at 37 °C. Western blot was used to analyse the samples as described above.

### Immunogenicity assay

Immunogenicity assays were carried out by Public Health England as follows: Sixty BALB/c mice aged 6–9 weeks were purchased from a Home Office‐approved breeder and supplier (Envigo). The mice were implanted with a temperature and identity chip during an acclimatization period. Groups of *n* = 6 mice (consisting of equal numbers of female and males) were immunized via the subcutaneous route in a single or a prime‐boost manner on day 0 and 14 with 100 µL of each candidate vaccine. Empty bluetongue CLPs and DENV1 EIII subunit were only analysed in a prime‐boost manner. For the VLP or CLP groups, the concentration was 10 µg/dose and for the subunit vaccine 0.7 µg/dose for comparable antigen molarity. 14 days after the last immunization, all mice were sacrificed and blood was collected for immunological analysis. Antibody response was measured using ELISA. To detect specific antibodies, 96‐well plates (NUNC Maxisorp) were coated with 5 µg/mL of each of the following antigens diluted in carbonate–bicarbonate buffer (Thermo, Product #28382): recombinant Zika virus envelope protein (Native Antigen Company, ZIKVSU‐ENV‐100), Zika VLP (Native Antigen Company, ZIKV‐VLP‐250), DENV1 VLP (Native Antigen Company, DENV1‐VLP‐250) and DENV4 VLP (Native Antigen Company, DENV4‐VLP‐250). Ten plates for each antigen were prepared and left overnight at 2–8 °C. Plates were then washed three times with PBS containing 0.05% (v/v) Tween‐20 (PBS‐Tween) and blocked with PBS containing 10% (w/v) skimmed milk powder for 2 h at room temperature. Serum samples were serially diluted twofold in PBS containing 2.5% (w/v) skimmed milk powder from 1:50 to 1:3200 before being added to the test plates in duplicate test wells. Control preparations of anti‐dengue mAb (Native Antigen Company, MAB12267‐500) and anti‐Zika mAb (Native Antigen Company, MAB12312‐500) were made starting at 10 µg/mL and then with 10‐fold dilutions to 0.01 µg/mL. Each ELISA plate had the control preparations in addition to 4 wells with no samples (diluent alone). Plates were incubated for 1 h at 37°C and following three washes with PBS‐Tween, 100 µL/well of peroxidase‐conjugated goat anti‐mouse IgG detector antibody diluted 1:5000 was added (Jackson ImmunoResearch, Product 115‐035‐071). Following incubation for 1 h at 37°C, plates were washed another three times with PBS‐Tween and peroxidase substrate was added (KPL TMB microwell peroxidase substrate system, Product 5120‐0047). After approximately 30 min, the reaction was halted by addition of stop solution (KPL TMB stop solution, Product 5420‐0026). Absorbances were read at a wavelength of 450 nm. Sample‐specific results were obtained by subtraction of the average absorbance from the 4 wells containing diluent only.

## Accession numbers

DENV Serotype 1 strain Hawaii: GenBank: KM204119.1

Serotype 2 strain New Guinea C: GenBank KM204118.1

Serotype 4 strain H241: GenBank KR011349.

Zika strain ZikaSPH2015: GenBank KU321639.

## Conflict of interest

G.P.L. declares that he is a named inventor on granted patent WO 29087391 A1, which describes the *HyperTrans* transient expression system used in this manuscript.

## Authors' contributions

DP, YM, ET, HP, ADA and RO designed constructs, and conducted expression tests in plants and purification and analysis of DENV1‐VLPs, BTV‐CLPs and DENV1‐EIII. SD and EK designed and conducted immunogenicity assays. HP, NH, MS and GPL supervised the work and gave scientific advice. All authors contributed to the manuscript.

## Supporting information


**Figure S1**Anti‐DENV E and anti‐prM Western blot of leaves co‐infiltrated with DENV‐SP and DENV‐NSP of serotype 1,2 and 4. Plants where harvested 6 dpi and analysed as described in material and methods. C = 100 ng DENV2‐VLPs positive control.
**Figure S2**Uncropped Western blots as shown in Figure 2 in the main text. A: Co‐expression of DENV1‐SP + DENV1‐NS3 (*) is not discussed in the original manuscript. No nonspecific bands or uncleaved polyproteins were detected. B: Membrane was cut after blotting and the upper part was analysed with anti‐DENV‐E and the lower part with anti‐prM antibodies.
**Figure S3**TEM analysis of purified bluetongue (BTV) core‐like particles (BTV c‐) displaying E domain III (EIII) of DENV1, DENV4 and Zika. Particles were negatively stained with 2% (w/v) uranyl acetate.
**Figure S4**Mass spectrometry analysis of B‐CLPs: The amino acid sequence of the ED3:VP3 fusion proteins was confirmed by mass spectrometry following a tryptic digest of gel‐purified protein. The presented data show an alignment of the N‐terminal E domain III sequences only. Green boxes show the sequences confirmed by presence of peptides in mass spectrometry. Homologous residues between DENV1, DENV4, and Zika EIII are shown in blue.
**Figure S5**Blots/gels of gradient fractions during purification of candidate vaccine VLPs. Samples were treated as described in material and method. Top: Fractions of the 3 different gradients used to purify DENV1 VLPs were analysed by anti‐E Western blotting, with the cleanest, most concentrated fractions (red stars) being pooled and taken forward to the next step. After the final sucrose gradient, the fractions indicated were pooled, buffer‐exchanged and concentrated as indicated in the Materials and Methods. Bottom: Fractions of the Optiprep gradients used to purify B‐CLPs were analysed by SDS‐PAGE with the cleanest fractions (red stars) being pooled and taken forward for buffer exchange and concentration as indicated in the Materials and Methods. The purification of the vaccine preparations involved numerous identical gradients being processed in parallel to increase production capacity, each of which was fractionated and analysed separately: representative blots and gels are shown here.
**Figure S6**Interferon‐gamma ELISPOT assays on splenocytes of vaccinated mice. An increased number of antigen‐specific IFN‐γ secreting cells to the peptides covering the ectodomain antigens were observed in animals immunized with the B‐CLP vaccine candidates under a prime‐boost approach, but not with a single immunization (6a). Statistical analysis using the non‐parametric Mann‐Whitney statistical test didn’t show any significant difference (*P* > 0.05) between these levels and that of the groups containing the bluetongue vector alone, likely due to the number of animals used (*n* = 6) and variability between animals. Peptide pools spanning the DENV1 E protein did not show any specific reactivity in the immunized animals (6b).
**Table S1** Used cloning primers: fw = forward, rv = reverse. Cloning sites: yellow = AgeI, green = XhoI, blue = BspEI, bold/underlined = NruI. Red sequence in F108A substitution of phenylalanine with alanine in DENV‐E.

## References

[pbi13501-bib-0001] Arora, U. , Tyagi, P. , Swaminathan, S. and Khanna, N. (2013) Virus‐like particles displaying envelope domain III of dengue virus type 2 induce virus‐specific antibody response in mice. Vaccine 31, 873–878. 23261049 10.1016/j.vaccine.2012.12.016

[pbi13501-bib-0002] Bachmann, M.F. and Jennings, G.T. (2010) Vaccine delivery: a matter of size, geometry, kinetics and molecular patterns. Nat. Rev. Immunol. 10, 787–796. 20948547 10.1038/nri2868

[pbi13501-bib-0003] Bachmann, M.F. and Zinkernagel, R.M. (1997) Neutralizing antiviral B cell responses. Annual Rev. Immunol. 15, 235–270. 9143688 10.1146/annurev.immunol.15.1.235

[pbi13501-bib-0004] Bhamarapravati, N. and Sutee, Y. (2000) Live attenuated tetravalent dengue vaccine. Vaccine 18, 44–47. 10821973 10.1016/s0264-410x(00)00040-2

[pbi13501-bib-0005] Bhatt, S. , Gething, P.W. , Brady, O.J. , Messina, J.P. , Farlow, A.W. , Moyes, C.L. , Drake, J.M. *et al*. (2013) The global distribution and burden of dengue. Nature 496, 504–507. 23563266 10.1038/nature12060 PMC3651993

[pbi13501-bib-0006] Boigard, H. , Alimova, A. , Martin, G.R. , Katz, A. , Gottlieb, P. and Galarza, J.M. (2017) Zika virus‐like particle (VLP) based vaccine. Plos Neglected Tropical Dis. 11, e0005608. 10.1371/journal.pntd.0005608 PMC5436897 28481898

[pbi13501-bib-0007] Chang, G.J.J. , Hunt, A.R. , Holmes, D.A. , Springfield, T. , Chiueh, T.‐S. , Roehrig, J.T. and Gubler, D.J. (2003) Enhancing biosynthesis and secretion of premembrane and envelope proteins by the chimeric plasmid of dengue virus type 2 and Japanese encephalitis virus. Virology 306, 170–180. 12620809 10.1016/s0042-6822(02)00028-4

[pbi13501-bib-0008] Charoensri, N. , Suphatrakul, A. , Sriburi, R. , Yasanga, T. , Junjhon, J. , Keelapang, P. , Utaipat, U. *et al*. (2014) An optimized expression vector for improving the yield of dengue virus‐like particles from transfected insect cells. J. Virol. Methods, 205, 116–123. 24814967 10.1016/j.jviromet.2014.04.019

[pbi13501-bib-0009] Charpilienne, A. , Nejmeddine, M. , Berois, M. , Parez, N. , Neumann, E. , Hewat, E. , Trugnan, G. *et al*. (2001) Individual rotavirus‐like particles containing 120 molecules of fluorescent protein are visible in living cells. J. Biol. Chem. 276, 29361–29367. 11356839 10.1074/jbc.M101935200

[pbi13501-bib-0010] Christenson, B. and Bottiger, M. (1994) Measles antibody: comparison of long‐term vaccination titres, early vaccination titres and naturally acquired immunity to and booster effects on the measles virus. Vaccine 12, 129–133. 8147093 10.1016/0264-410x(94)90049-3

[pbi13501-bib-0011] D'Aoust, M.A. , Couture, M.M. , Charland, N. , Trepanier, S. , Landry, N. , Ors, F. & Vezina, L.P. (2010) The production of hemagglutinin‐based virus‐like particles in plants: a rapid, efficient and safe response to pandemic influenza. Plant Biotechnol. J. 8, 607–619. 20199612 10.1111/j.1467-7652.2009.00496.x

[pbi13501-bib-0012] D'Aoust, M.A. , Lavoie, P.O. , Couture, M.M. , Trepanier, S. , Guay, J.M. , Dargis, M. , Mongrand, S. *et al*. (2008) Influenza virus‐like particles produced by transient expression in Nicotiana benthamiana induce a protective immune response against a lethal viral challenge in mice. Plant Biotechnol. J. 6, 930–940. 19076615 10.1111/j.1467-7652.2008.00384.x

[pbi13501-bib-0013] De Alwis, R. , Smith, S.A. , Olivarez, N.P. , Messer, W.B. , Huynh, J.P. , Wahala, W.M.P.B. , White, L.J. *et al*. (2012) Identification of human neutralizing antibodies that bind to complex epitopes on dengue virions. Proc. Natl. Acad. Sci. USA, 109, 7439–7444. 22499787 10.1073/pnas.1200566109 PMC3358852

[pbi13501-bib-0014] Fay, P.C. (2018) Antigenic and phylogenetic relationships of outer capsid protein VP2, from multiple bluetongue virus serotypes. PhD Thesis, University of Nottingham. http://eprints.nottingham.ac.uk/id/eprint/52145

[pbi13501-bib-0015] Fuenmayor, J. , Godia, F. and Cervera, L. (2017) Production of virus‐like particles for vaccines. N. Biotechnol. 39, 174–180. 28778817 10.1016/j.nbt.2017.07.010 PMC7102714

[pbi13501-bib-0016] Galula, J.U. , Shen, W.F. , Chuang, S.T. , Chang, G.J.J. and Chao, D.Y. (2014) Virus‐like particle secretion and genotype‐dependent immunogenicity of dengue virus serotype 2 DNA vaccine. J. Virol. 88, 10813–10830. 25008922 10.1128/JVI.00810-14 PMC4178884

[pbi13501-bib-0017] Garg, H. , Sedano, M. , Plata, G. , Punke, E.B. and Joshi, A. (2017) Development of virus‐like‐particle vaccine and reporter assay for Zika virus. J. Virol. 91, e00834‐17. 28794019 10.1128/JVI.00834-17 PMC5625514

[pbi13501-bib-0018] Gottschamel, J. , Lossl, A. , Ruf, S. , Wang, Y.L. , Skaugen, M. , Bock, R. and Clarke, J.L. (2016) Production of dengue virus envelope protein domain III‐based antigens in tobacco chloroplasts using inducible and constitutive expression systems. Plant Mol. Biol. 91, 497–512. 27116001 10.1007/s11103-016-0484-5

[pbi13501-bib-0019] Halstead, S.B. (2007) Dengue. Lancet 370, 1644–1652. 17993365 10.1016/S0140-6736(07)61687-0

[pbi13501-bib-0020] Hasan, S.S. , Sevvana, M. , Kuhn, R.J. and Rossmann, M.G. (2018) Structural biology of Zika virus and other flaviviruses. Nat. Struct. Mol. Biol. 25, 13–20. 29323278 10.1038/s41594-017-0010-8

[pbi13501-bib-0021] Hsieh, S.C. , Liu, I.J. , King, C.C. , Chang, G.J. and Wang, W.K. (2008) A strong endoplasmic reticulum retention signal in the stem‐anchor region of envelope glycoprotein of dengue virus type 2 affects the production of virus‐like particles. Virology 374, 338–350. 18252258 10.1016/j.virol.2007.12.041

[pbi13501-bib-0022] Kanagaraj, A.P. , Verma, D. and Daniell, H. (2011) Expression of dengue‐3 premembrane and envelope polyprotein in lettuce chloroplasts. Plant Mol. Biol. 76, 323–333. 21431782 10.1007/s11103-011-9766-0 PMC3468899

[pbi13501-bib-0023] Kongsgaard, M. , Bassi, M.R. , Rasmussen, M. , Skjodt, K. , Thybo, S. , Gabriel, M. , Hansen, M.B. *et al*. (2017) Adaptive immune responses to booster vaccination against yellow fever virus are much reduced compared to those after primary vaccination. Sci. Rep. 7, 662. 28386132 10.1038/s41598-017-00798-1 PMC5429613

[pbi13501-bib-0024] Li, L. , Lok, S.M. , Yu, I‐m. , Zhang, Y. , Kuhn, R.j. , Chen, J. and Rossmann, M.g .*et al*. (2008) The flavivirus precursor membrane‐envelope protein complex: structure and maturation. Science 319, 1830–1834. 18369147 10.1126/science.1153263

[pbi13501-bib-0025] Lico, C. , Santi, L. , Twyman, R.M. , Pezzotti, M. and Avesani, L. (2012) The use of plants for the production of therapeutic human peptides. Plant Cell Rep. 31, 439–451. 22218674 10.1007/s00299-011-1215-7

[pbi13501-bib-0026] Lindenbach, B.D. , Thiel, H.J. , Rice, C.M. (2007) Flaviviridae: The viruses and their replication. In Fields Virology( Knipe, D.M. , and Howley, P.M. ed.). Philadelphia: Lippincott‐Raven Publishers.

[pbi13501-bib-0027] Link, A. , Zabel, F. , Schnetzler, Y. , Titz, A. , Brombacher, F. and Bachmann, M.F. (2012) Innate immunity mediates follicular transport of particulate but not soluble protein antigen. J. Immunol. 188, 3724–3733. 22427639 10.4049/jimmunol.1103312

[pbi13501-bib-0028] Lomonossoff, G.P. and D'Aoust, M. A. (2016) Plant‐produced biopharmaceuticals: A case of technical developments driving clinical deployment. Science 353, 1237–1240. 27634524 10.1126/science.aaf6638

[pbi13501-bib-0029] Ma, J.K.C. , Barros, E. , Bock, R. , Christou, P. , Dale, P.J. , Dix, P.J. , Fischer, R. *et al*. (2005) Molecular farming for new drugs and vaccines ‐ Current perspectives on the production of pharmaceuticals in transgenic plants. EMBO Rep. 6, 593–599. 15995674 10.1038/sj.embor.7400470 PMC1369121

[pbi13501-bib-0030] Ma, J.K.C. , Drake, P.M.W. and Christou, P. (2003) The production of recombinant pharmaceutical proteins in plants. Nat. Rev. Genet. 4, 794–805. 14526375 10.1038/nrg1177

[pbi13501-bib-0031] Mamedov, T. , Musayeva, I. , Acsora, R. , Gun, N. , Gulec, B. , Mammadova, G. , Cicek, K. *et al*. (2019) Engineering, and production of functionally active human Furin in N. benthamiana plant: In vivo post‐translational processing of target proteins by Furin in plants. PLoS One 14, e0213438. 30861020 10.1371/journal.pone.0213438 PMC6413912

[pbi13501-bib-0032] Mani, S. , Tripathi, L. , Raut, R. , Tyagi, P. , Arora, U. , Barman, T. , Sood, R. *et al*. (2013) Pichia pastoris‐expressed dengue 2 envelope forms virus‐like particles without pre‐membrane protein and induces high titer neutralizing antibodies. PLoS One 8, e64595. 23717637 10.1371/journal.pone.0064595 PMC3662778

[pbi13501-bib-0033] Marsian, J. (2016) Transient expression of poliovirus‐like particles in plants. Developing a synthetic polio vaccine. PhD Thesis, University of East Anglia. https://ueaeprints.uea.ac.uk/id/eprint/62929

[pbi13501-bib-0034] Marsian, J. , Fox, H. , Bahar, M.W. , Kotecha, A. , Fry, E.E. , Stuart, D.I. , Macadam, A.J. *et al*. (2017) Plant‐made polio type 3 stabilized VLPs‐a candidate synthetic polio vaccine. Nat. Commun. 8, 245. 28811473 10.1038/s41467-017-00090-w PMC5557999

[pbi13501-bib-0035] Marsian, J. , Hurdiss, D.L. , Ranson, N.A. , Ritala, A. , Paley, R. , Cano, I. and Lomonossoff, G.P. (2019) Plant‐made nervous necrosis virus‐like particles protect fish against disease. Front. Plant Sci. 10, 880. 31354759 10.3389/fpls.2019.00880 PMC6629939

[pbi13501-bib-0036] Mason, P.W. , Pincus, S. , Fournier, M.J. , Mason, T.L. , Shope, R.E. and Paoletti, E. (1991) Japanese encephalitis virus‐vaccinia recombinants produce particulate forms of the structural membrane proteins and induce high levels of protection against lethal JEV infection. Virology 180, 294–305. 1845826 10.1016/0042-6822(91)90034-9

[pbi13501-bib-0037] Mathew, L.G. , Herbst‐Kralovetz, M.M. and Mason, H.S. (2014) Norovirus Narita 104 virus‐like particles expressed in *Nicotiana benthamiana* induce serum and mucosal immune responses. Biomed. Res. Int. 2014, Article ID 807539. (PMID: 24949472). 24949472 10.1155/2014/807539 PMC4037605

[pbi13501-bib-0038] Mbewana, S. , Meyers, A.E. and Rybicki, E.P. (2019) Chimaeric rift valley fever virus‐like particle vaccine candidate production in *Nicotiana benthamiana* . Biotechnol. J. 14, e1800238. 30488669 10.1002/biot.201800238

[pbi13501-bib-0039] Mechtcheriakova, I.A. , Eldarov, M.A. , Nicholson, L. , Shanks, M. , Skryabin, K.G. and Lomonossoff, G.P. (2006) The use of viral vectors to produce hepatitis B virus core particles in plants. J. Virol. Methods, 131, 10–15. 16112207 10.1016/j.jviromet.2005.06.020

[pbi13501-bib-0040] Metz, S.W. , Thomas, A. , White, L. , Stoops, M. , Corten, M. , Hannemann, H. and De Silva, A.M. (2018) Dengue virus‐like particles mimic the antigenic properties of the infectious dengue virus envelope. Virol. J. 15, 60. 10.1186/s12985-018-0970-2 PMC5879749 29609659

[pbi13501-bib-0041] Mondotte, J.A. , Lozach, P.Y. , Amara, A. and Gamarnik, A.V. (2007) Essential role of dengue virus envelope protein N glycosylation at asparagine‐67 during viral propagation. J. Virol. 81, 7136–7148. 17459925 10.1128/JVI.00116-07 PMC1933273

[pbi13501-bib-0042] Pang, E.L. , Peyret, H. , Ramirez, A. , Loh, H.S. , Lai, K.S. , Fang, C.M. , Rosenberg, W.M. *et al*. (2019) Epitope presentation of dengue viral envelope glycoprotein domain III on Hepatitis B core protein virus‐like particles produced in *Nicotiana benthamiana* . Front Plant Sci. 10, 455. 31057572 10.3389/fpls.2019.00455 PMC6477658

[pbi13501-bib-0043] Park, J. , Lee, H.Y. , Khai, L.T. , Thuy, N.T.T. , Mai, L.Q. and Jang, Y.S. (2020) Addition of partial envelope domain II into envelope domain III of dengue virus antigen potentiates the induction of virus‐neutralizing antibodies and induces protective immunity. Vaccines (Basel), 8, 88. 32075300 10.3390/vaccines8010088 PMC7157711

[pbi13501-bib-0044] Peyret, H. , Gehin, A. , Thuenemann, E.C. , Blond, D. , El Turabi, A. , Beales, L. , Clarke, D. *et al*. (2015) Tandem fusion of hepatitis B core antigen allows assembly of virus‐like particles in bacteria and plants with enhanced capacity to accommodate foreign proteins. PLoS One 10, e0120751. 25830365 10.1371/journal.pone.0120751 PMC4382129

[pbi13501-bib-0045] Pincus, S. , Mason, P.W. , Konishi, E. , Fonseca, B.A. , Shope, R.E. , Rice, C.M. and Paoletti, E. (1992) Recombinant vaccinia virus producing the prM and E proteins of yellow fever virus protects mice from lethal yellow fever encephalitis. Virology 187, 290–297. 1736531 10.1016/0042-6822(92)90317-i

[pbi13501-bib-0046] Ramasamy, V. , Arora, U. , Shukla, R. , Poddar, A. , Shanmugam, R.K. , White, L.J. , Mattocks, M.M. *et al*. (2018) A tetravalent virus‐like particle vaccine designed to display domain III of dengue envelope proteins induces multi‐serotype neutralizing antibodies in mice and macaques which confer protection against antibody dependent enhancement in AG129 mice. PLoS Negl. Trop Dis. 12, e0006191. 29309412 10.1371/journal.pntd.0006191 PMC5774828

[pbi13501-bib-0047] Rodenhuis‐Zybert, I.A. , Wilschut, J. and Smit, J.M. (2010) Dengue virus life cycle: viral and host factors modulating infectivity. Cell Mol. Life Sci. 67, 2773–2786. 20372965 10.1007/s00018-010-0357-z PMC11115823

[pbi13501-bib-0048] Sainsbury, F. , Thuenemann, E.C. and Lomonossoff, G.P. (2009) pEAQ: versatile expression vectors for easy and quick transient expression of heterologous proteins in plants. Plant Biotechnol. J. 7, 682–693. 19627561 10.1111/j.1467-7652.2009.00434.x

[pbi13501-bib-0049] Smith, S.A. , Nivarthi, U.K. , De Alwis, R. , Kose, N. , Sapparapu, G. , Bombardi, R. and Kahle, K.M. *et al*. (2016) Dengue virus prM‐specific human monoclonal antibodies with virus replication‐enhancing properties recognize a single immunodominant antigenic site. J. Virol. 90, 780–9. 26512092 10.1128/JVI.01805-15 PMC4702676

[pbi13501-bib-0050] Sugrue, R.J. , Fu, J.L. , Howe, J. and Chan, Y.C. (1997) Expression of the dengue virus structural proteins in Pichia pastoris leads to the generation of virus‐like particles. J. Gen. Virol. 78, 1861–1866. 9266980 10.1099/0022-1317-78-8-1861

[pbi13501-bib-0051] Takahashi, H. , Ohtaki, N. , Maeda‐Sato, M. , Tanaka, M. , Tanaka, K. , Sawa, H. , Ishikawa, T. *et al*. (2009) Effects of the number of amino acid residues in the signal segment upstream or downstream of the NS2B‐3 cleavage site on production and secretion of prM/M‐E virus‐like particles of West Nile virus. Microbes Infect. 11, 1019–1028. 19647801 10.1016/j.micinf.2009.07.009

[pbi13501-bib-0052] Thuenemann, E.C. , Meyers, A.E. , Verwey, J. , Rybicki, E.P. and Lomonossoff, G.P. (2013) A method for rapid production of heteromultimeric protein complexes in plants: assembly of protective bluetongue virus‐like particles. Plant Biotechnol. J. 11, 839–846. 23647743 10.1111/pbi.12076

[pbi13501-bib-0053] Twyman, R.M. , Stoger, E. , Schillberg, S. , Christou, P. and Fischer, R. (2003) Molecular farming in plants: host systems and expression technology. Trends Biotechnol. 21, 570–578. 14624867 10.1016/j.tibtech.2003.10.002

[pbi13501-bib-0054] Urakami, A. , Ngwe Tun, M.M. , Moi, M.L. , Sakurai, A. , Ishikawa, M. , Kuno, S. , Ueno, R. *et al*. (2017) An envelope‐modified tetravalent dengue virus‐like‐particle vaccine has implications for flavivirus vaccine design. J. Virol. 91(23). 10.1128/jvi.01181-17 PMC5686733 28956764

[pbi13501-bib-0055] Wahala, W.M. , Kraus, A.A. , Haymore, L.B. , Accavitti‐Loper, M.A. and De Silva, A.M. (2009) Dengue virus neutralization by human immune sera: role of envelope protein domain III‐reactive antibody. Virology 392, 103–113. 19631955 10.1016/j.virol.2009.06.037 PMC2746956

[pbi13501-bib-0056] WHO (2018). Dengue vaccine: WHO position paper – September 2018. Wkly Epidemiol. Rec. 36, 96, 457–476.

[pbi13501-bib-0057] Wilbers, R.H.P. , Westerhof, L.B. , Van Raaij, D.R. , Van Adrichem, M. , Prakasa, A.D. , Lozano‐Torres, J.L. , Bakker, J. *et al*. (2016) Co‐expression of the protease furin in Nicotiana benthamiana leads to efficient processing of latent transforming growth factor‐1 into a biologically active protein. Plant Biotechnol. J. 14, 1695–1704. 26834022 10.1111/pbi.12530 PMC5067602

[pbi13501-bib-0058] Xie, X. , Zou, J. , Zhang, X. , Zhou, Y. , Routh, A.L. , Kang, C. , Popov, V.L. *et al*. (2019) Dengue NS2A protein orchestrates virus assembly. Cell Host Microbe, 26, 606–622, e8. 31631053 10.1016/j.chom.2019.09.015

[pbi13501-bib-0059] Zhang, S. , Liang, M.F. , Gu, W. , Li, C. , Miao, F. , Wang, X.F. , Jin, C. *et al*. (2011) Vaccination with dengue virus‐like particles induces humoral and cellular immune responses in mice. Virol. J. 8(1), 333. 10.1186/1743-422x-8-333 21714940 PMC3144018

