# Supplementary material for: Plant‐made dengue virus‐like particles produced by co‐expression of structural and non‐structural proteins induce a humoral immune response in mice
Source: Plant Biotechnol J. 2020 Nov 22;19(4):745–56. doi: 10.1111/pbi.13501 (PMC8051607; doi:10.1111/pbi.13501)
Supplement: Supplementary file 1 — Figure S1Anti‐DENV E and anti‐prM Western blot of leaves co‐infiltrated with DENV‐SP and DENV‐NSP of serotype 1,2 and 4. Plants where harvested 6 dpi and analysed as described in material and methods. C = 100 ng DENV2‐VLPs positive control. Figure S2Uncropped Western blots as shown in Figure 2 in the main text. A: Co‐expression of DENV1‐SP + DENV1‐NS3 (*) is not discussed in the original manuscript. No nonspecific bands or uncleaved polyproteins were detected. B: Membrane was cut after blotting and the upper part was analysed with anti‐DENV‐E and the lower part with anti‐prM antibodies. Figure S3TEM analysis of purified bluetongue (BTV) core‐like particles (BTV c‐) displaying E domain III (EIII) of DENV1, DENV4 and Zika. Particles were negatively stained with 2% (w/v) uranyl acetate. Figure S4Mass spectrometry analysis of B‐CLPs: The amino acid sequence of the ED3:VP3 fusion proteins was confirmed by mass spectrometry following a tryptic digest of gel‐purified protein. The presented data show an alignment of the N‐terminal E domain III sequences only. Green boxes show the sequences confirmed by presence of peptides in mass spectrometry. Homologous residues between DENV1, DENV4, and Zika EIII are shown in blue. Figure S5Blots/gels of gradient fractions during purification of candidate vaccine VLPs. Samples were treated as described in material and method. Top: Fractions of the 3 different gradients used to purify DENV1 VLPs were analysed by anti‐E Western blotting, with the cleanest, most concentrated fractions (red stars) being pooled and taken forward to the next step. After the final sucrose gradient, the fractions indicated were pooled, buffer‐exchanged and concentrated as indicated in the Materials and Methods. Bottom: Fractions of the Optiprep gradients used to purify B‐CLPs were analysed by SDS‐PAGE with the cleanest fractions (red stars) being pooled and taken forward for buffer exchange and concentration as indicated in the Materials and Methods. The purific [file PBI-19-745-s001.pdf]

Plant-made Dengue virus-like particles produced by co-expression of structural and non-structural proteins induce a humoral immune response in mice.

Daniel Ponndorf[1], Yulia Meshcheriakova[1], Eva Thuenemann[1], Albor Dobon Alonso[2], Ross Overman[2], Nicholas Holton[2], Stuart Dowall[3], Emma Kennedy[3], Martin Stocks[4], George Lomonossoff[1], Hadrien Peyret[1*]

Supplemental material

Table S 1: Used cloning primers: fw = forward, rv = reverse. Cloning sites: yellow = AgeI, green = XhoI, blue = BspEI, bold/underlined = NruI. Red sequence in F108A substitution of phenylalanine with alanine in DENV-E.

Primer name	Sequence
DENV1-SP-fw	GACGACACCGGTAACAATGAACAACCAGAG
DENV1-SP-rv	GTTGTTCTCGAGCTAAGCCTGCACCATAAC
DENV1-NSP-fw	TACTATACCGGTAACAATGGATTCTGGTTGCGTGATCAAC
DENV1-NSP-rv	ATAATACTCGAGCTACTTCCACTTCTCTCCAAGG
DENV1-PrM-E-fw	CCTTACCGGTAACAATGCTTCTTATGCTGCTGCCTAC
DENV1-PrM-E-rv	GAAACTCGAGCTAAGCCTGCACCATAACACCCAG
DENV1-F108A-fw	GATGGGGTAACGGTTGCGGTCTTGCTGGTAAGGGTTCTCTGATTA
DENV1-F108A-rv	TAATCAGAGAACCCTTACCAGCAAGACCGCAACCGTTACCCCATC
ZIKV-PrM-E-12-fw	GATTACCGGTAACAATGACCTCCGTTGGTATTGTGGG
ZIKV-PrM-E-12-rv	CAAACTCGAGCTAATCAGCAGACACAGCGGTGC
DENV1-EIII-fw	AAAT**TCGCGA**CCGGTATGAAGGGCATGAGCTACGTGATG
DENV1-EIII-rv	TGCCTCCGGACTTCTTGAACCAGCTAAGCTTCAGTG
DENV4-EIII-fw	AAAT**TCGCGA**CCGGTATGAAGGGCATGAGCTACACCATG
DENV4-EIII-rv	TGCCTCCGGACTTCCTAAACCAGTGAAGGGTCAG
ZIKV-EIII-fw	AAAT**TCGCGA**CCGGT ATGGCTTTTACCTTCACCAAGATCCC
ZZIKV-EIII-rv	TGCCTCCGGAGGACCTGTGCCAGTGATGAG
DENV1_EIII - fw	CTCTCAGTTCAGCTGAAAAGGGCATGAGCTACGTG
DENV1_EIII - rv	GTGATGGTGACCACTACCCTTCTTGAACCAGCTAAGC
SP-Atl-fw	TAAGCAACCGGTATAAACAATGAAAACTAACCTCTTTCTCTTTCTAATCTTCTCACTTCTGCTTTCTCTCAGTTCAGCTGAA
His-Spytag- rv	TGCTTACTCGAGTTATTTAGTAGGCTTGTACGCATCTACCATTACGATGTGAGCTCCAGACCCATGATGATGGTGATGGTGACCACTACC

Figure S1

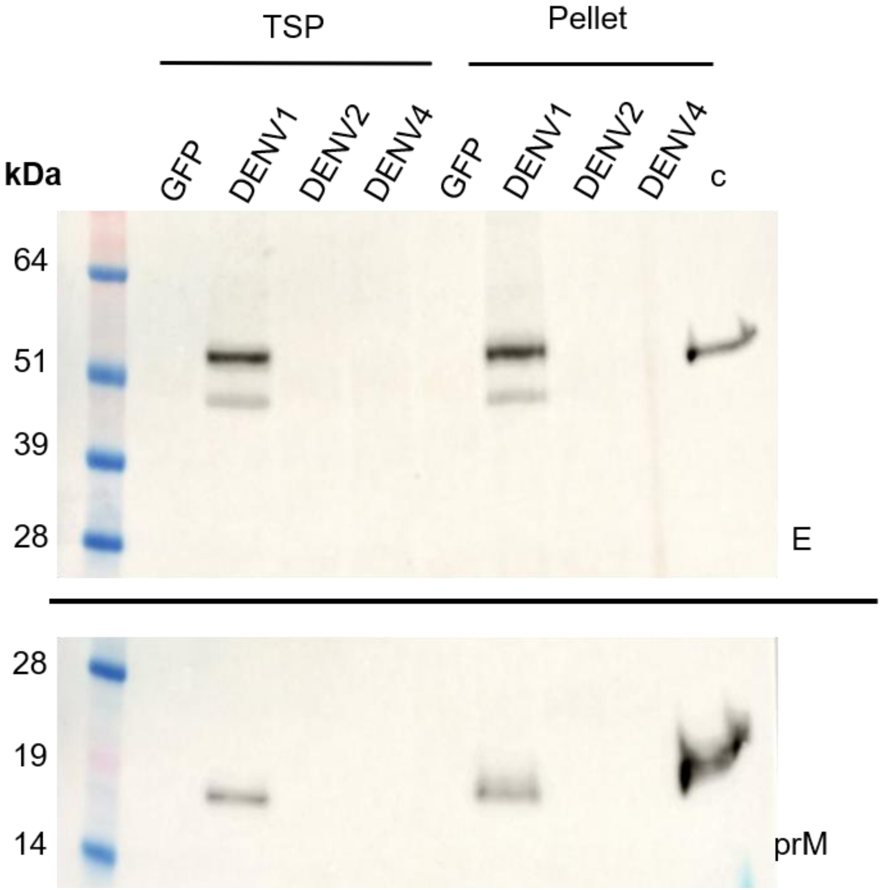

Figure S 1: Anti-DENV E and anti-prM Western blot of leaves co-infiltrated with DENV-SP and DENV-NSP of serotype 1,2 and 4. Plants where harvested 6 dpi and analysed as described in material and methods. C=100ng DENV2-VLPs positive control.

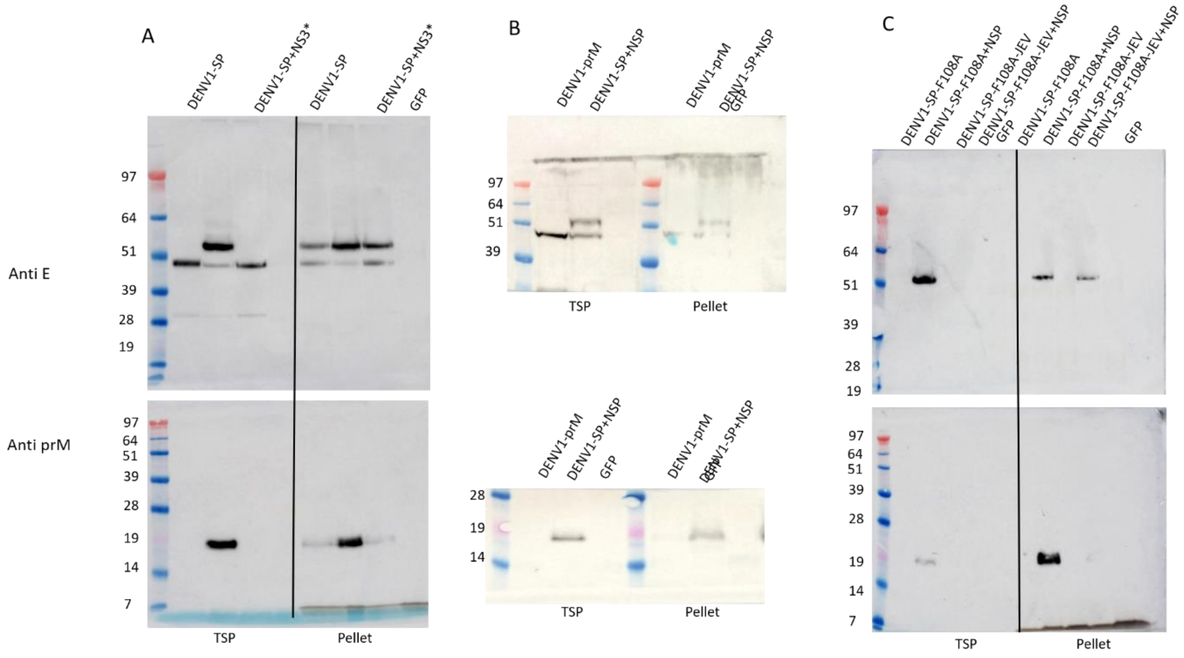

Figure S 2 Uncropped Western blots as shown in Figure 2 in the main text. A: Co-expression of DENV1-SP+DENV1-NS3 (*) is not discussed in the original manuscript. No non-specific bands or uncleaved polyproteins where detected. B: Membrane was cut after blotting and the upper part was analysed with anti DENV-E- and the lower part with anti-prM antibodies.

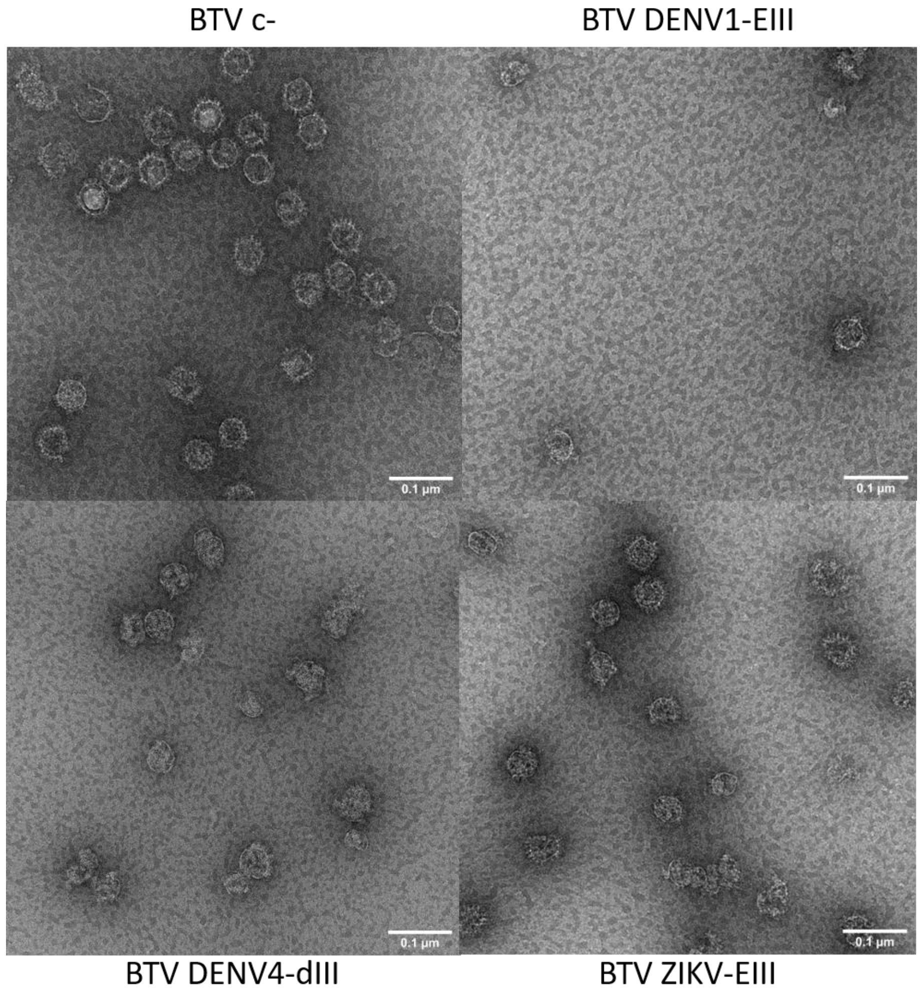

Figure S 3: TEM analysis of purified bluetongue (BTV) core-like particles (BTV c-) displaying E domain III (EIII) of DENV1, DENV4 and Zika. Particles were negatively stained with 2% (w/v) uranyl acetate.

```
pEAQ-HT-DENV1-EIII-BTV   MKGMSYVMCT GSFKLEKEVA ETQHGTVLVQ VKYEGTDAPC KIPFSTQDEK GVTQ-NGRLI TANPIVTDKE --KPVNIEAE PPFGESYIVV GAGEKALKLS WFKKSGG
pEAQ-HT-DENV4-EIII-BTV   MKGMSYTMCS GKFSIDKEMA ETQHGTTVVK VKYEGAGAPC KVPIEIRDVN KEKV-VGRII SSTPFAEYTN --SVTNIELE PPFGDSYIVI GVGDSALTLH WFRKSGG
pEAQ-HT-ZIKA-EIII-BTV    ---------- MAFTFTKIPA ETLHGTVTVE VQYAGTDGPC KVPAQMAVDM QTLTPVGRLI TANPVITEST ENSKMMLELD PPFGDSYIVI GVGEKKITHH WHRSSGG
```

Figure S 4 Mass spectrometry analysis of B-CLPs: The amino acid sequence of the ED3:VP3 fusion proteins was confirmed by mass spectrometry following a tryptic digest of gel-purified protein. The presented data show an alignment of the N-terminal E domain III sequences only. Green boxes show the sequences confirmed by presence of peptides in mass spectrometry. Homologous residues between DENV1, DENV4, and Zika EIII are shown in blue.

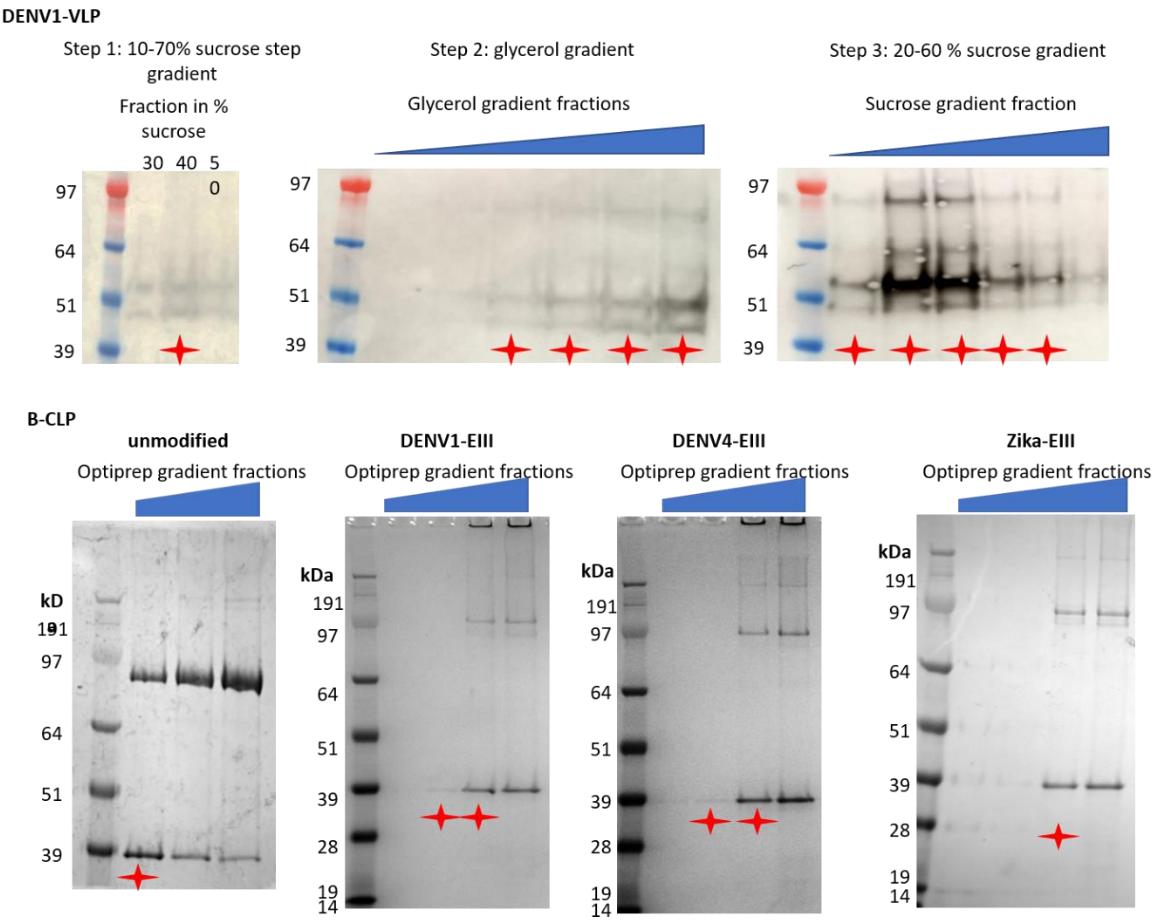

Figure S 5: Blots/gels of gradient fractions during purification of candidate vaccine VLPs. Samples were treated as described in material and method. Top: Fractions of the 3 different gradients used to purify DENV1 VLPs were analysed by anti-E Western blotting, with the cleanest, most concentrated fractions (red stars) being pooled and taken forward to the next step. After the final sucrose gradient, the fractions indicated were pooled, buffer exchanged and concentrated as indicated in the Materials and Methods. Bottom: Fractions of the optiprep gradients used to purify B-CLPs were analysed by SDS-PAGE with the cleanest fractions (red stars) being pooled and taken forward for buffer exchange and concentration as indicated in the Materials and Methods. The purification of the vaccine preparations involved numerous identical gradients being processed in parallel to increase production capacity, each of which was fractionated and analysed separately: representative blots and gels are shown here.

Figure S 6 – Interferon-gamma ELISPOT assays on splenocytes of vaccinated mice. Interferon-gamma ELISPOT assays were conducted using a commercial kit (Mouse IFN-γ ELISPOT plus, MabTech, Product 3321-4APW-10, Batch 74). To each well, $2x10^5$ splenocytes were added along with peptides pools (JPT peptides). Peptides were 15 amino acids long and overlapped by 11 amino acids covering the Dengue 1 E (121 peptides in 11 pools), Dengue 1 ectodomain (22 peptides in 2 pools), Dengue 4 ectodomain (22 peptides in 2 pools) and Zika ectodomain III (21 peptides in 2 pools) using sequences sent by the John Innes Centre. Peptides were tested at a concentration of 25µg/ml per each individual peptide in the pool. Each peptide pool was tested in duplicate for each animal alongside 13 negative wells with no stimulation and 1 positive control well containing stimulation media. After overnight incubation at 37°C, plates were developed to produce staining around cells secreting IFN-g. Spots were counted on an automated ELISPOT reader (Cellular Technologies Ltd). Background values were subtracted from test wells and summed for the pools across the target protein. Data were expressed as spot-forming cells per 10^6 cells.

An increased number of antigen-specific IFN-γ secreting cells to the peptides covering the ectodomain antigens were observed in animals immunised with the B-CLP vaccine candidates under a prime-boost approach, but not with a single immunisation (Figure S 6a). Statistical analysis using the non-parametric Mann-Whitney statistical test didn't show any significant difference (P>0.05) between these levels and that of the groups containing the bluetongue vector alone, likely due to the number of animals used (n=6) and variability between animals. Peptide pools spanning the DENV1 E protein did not show any specific reactivity in the immunised animals (Figure S 6b).

A

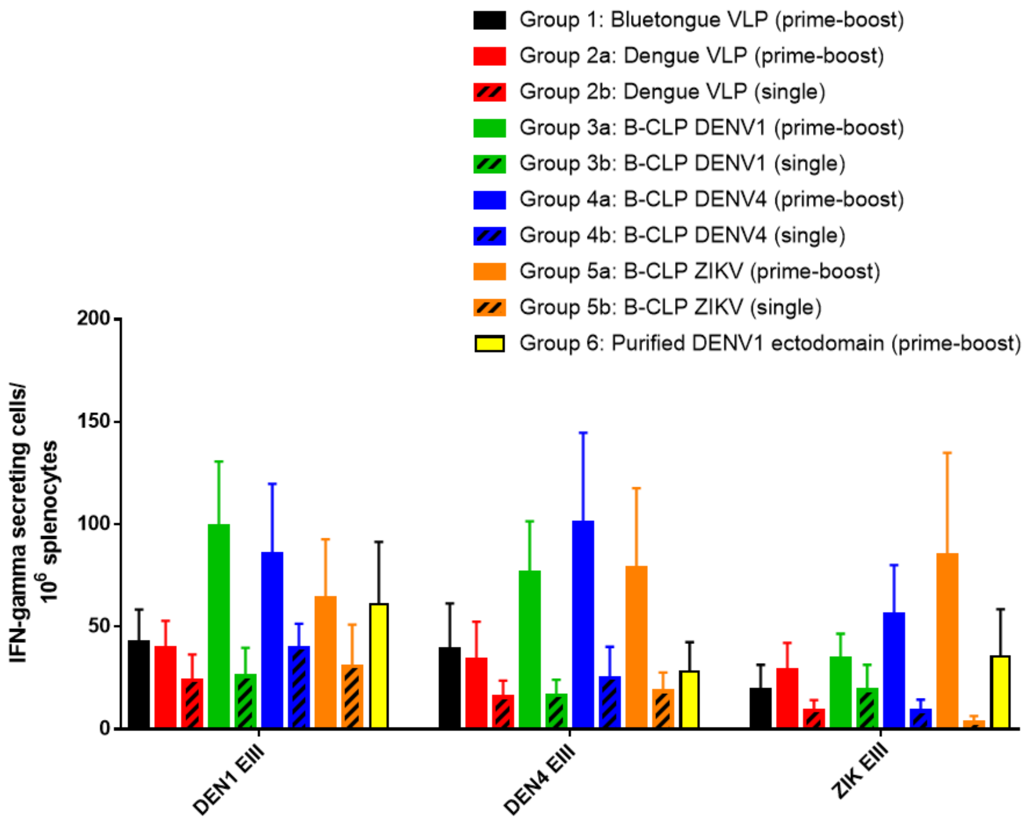

Figure S 6a: Frequency of antigen-specific IFN-g secreting cells to peptide pools covering DENV1, DENV4 and ZIKV ectodomain in splenocytes from animals immunised with vaccine candidates.

B

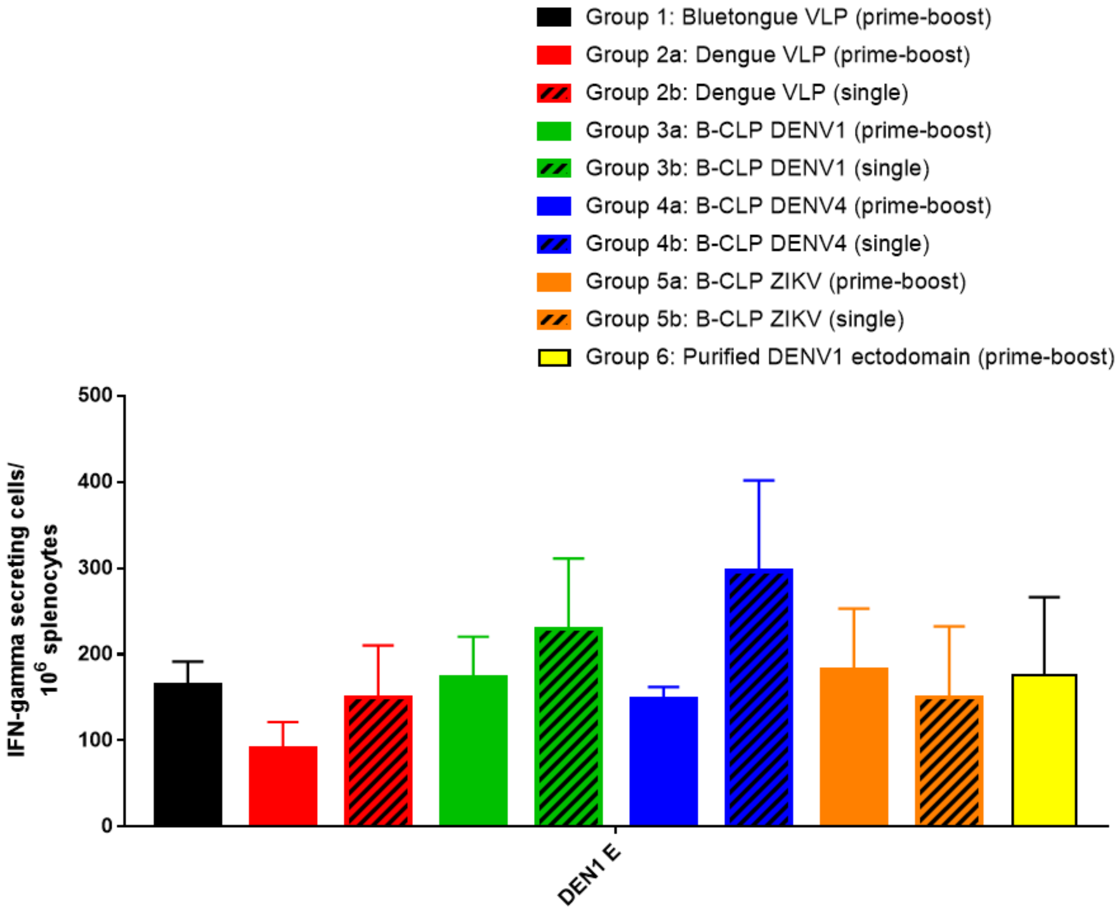

Figure S 6b: Frequency of antigen-specific IFN-g secreting cells to peptide pools covering the DENV1, E protein in splenocytes from animals immunised with vaccine candidates.
